# The effect of disease co-occurrence measurement on multimorbidity networks: a population-based study

**DOI:** 10.1186/s12874-022-01607-8

**Published:** 2022-06-08

**Authors:** Barret A. Monchka, Carson K. Leung, Nathan C. Nickel, Lisa M. Lix

**Affiliations:** 1 Department of Community Health Sciences, University of Manitoba, Winnipeg, Manitoba Canada; 2 George and Fay Yee Centre for Healthcare Innovation, University of Manitoba, 3rd Floor, 753 McDermot Ave, Winnipeg, Manitoba R3E 0T6 Canada; 3 Department of Computer Science, University of Manitoba, Winnipeg, Manitoba Canada; 4 Manitoba Centre for Health Policy, University of Manitoba, Winnipeg, Manitoba Canada

**Keywords:** Administrative health data, Association rule mining, Chronic disease, Disease co-occurrence, Multimorbidity, Network analysis

## Abstract

**Background:**

Network analysis, a technique for describing relationships, can provide insights into patterns of co-occurring chronic health conditions. The effect that co-occurrence measurement has on disease network structure and resulting inferences has not been well studied. The purpose of the study was to compare structural differences among multimorbidity networks constructed using different co-occurrence measures.

**Methods:**

A retrospective cohort study was conducted using four fiscal years of administrative health data (2015/16 – 2018/19) from the province of Manitoba, Canada (population 1.5 million). Chronic conditions were identified using diagnosis codes from electronic records of physician visits, surgeries, and inpatient hospitalizations, and grouped into categories using the Johns Hopkins Adjusted Clinical Group (ACG) System. Pairwise disease networks were separately constructed using each of seven co-occurrence measures: lift, relative risk, phi, Jaccard, cosine, Kulczynski, and joint prevalence. Centrality analysis was limited to the top 20 central nodes, with degree centrality used to identify potentially influential chronic conditions. Community detection was used to identify disease clusters. Similarities in community structure between networks was measured using the adjusted Rand index (ARI). Network edges were described using disease prevalence categorized as low (< 1%), moderate (1 to < 7%), and high (≥7%). Network complexity was measured using network density and frequencies of nodes and edges.

**Results:**

Relative risk and lift highlighted co-occurrences between pairs of low prevalence health conditions. Kulczynski emphasized relationships between high and low prevalence conditions. Joint prevalence focused on highly-prevalent conditions. Phi, Jaccard, and cosine emphasized associations involving moderately prevalent conditions. Co-occurrence measurement differences significantly affected the number and structure of identified disease clusters. When limiting the number of edges to produce visually interpretable graphs, networks had significant dissimilarity in the percentage of co-occurrence relationships in common, and in their selection of the highest-degree nodes.

**Conclusions:**

Multimorbidity network analyses are sensitive to disease co-occurrence measurement. Co-occurrence measures should be selected considering their intrinsic properties, research objectives, and the health condition prevalence relationships of greatest interest. Researchers should consider conducting sensitivity analyses using different co-occurrence measures.

**Supplementary Information:**

The online version contains supplementary material available at 10.1186/s12874-022-01607-8.

## Background

Multimorbidity, the co-existence of two or more chronic health conditions within an individual, where none are considered more central than the others, is becoming increasingly common in populations worldwide [1–3]. Those living with multiple chronic conditions tend to experience poorer quality of life, have increased disability and mortality, and face many challenges accessing healthcare services including receiving conflicting medical advice and duplicative testing, experiencing drug interactions, and managing a heavy treatment burden [3–6]. Multimorbidity also places a strain on healthcare systems since individuals with multiple chronic conditions have higher healthcare utilization and costs [7–9].

Network analysis, the study of relationships amongst connected entities, is commonly used to examine social relationships but has only recently been proposed as a method to shed light on population-level multimorbidity patterns. Network analysis models disease co-occurrence using graph structures characterized by nodes (e.g., diseases) and connecting edges (i.e., relationships or interactions). Several recent studies applied network analysis to electronic health data, to examine associations among co-occurring health conditions at the population level [10–25]. Network analysis is appealing for multimorbidity research, in part because of its reliance on graphical techniques to present disease associations, which can efficiently convey information in a non-technical manner to clinicians, patients, and healthcare system decision makers. Along with data visualization, network analysis also supports 1) centrality analysis for the detection of important nodes or hubs, that is, diseases that may be influential in a population or among a set of other diseases; 2) the identification of community structure, that is, clusters of highly-connected diseases; and 3) comparisons between population subgroups by contrasting network properties such as density and complexity measures. Although the practical implications of multimorbidity network analyses have not been fully explored, data-driven patient profiles built using network analysis can be used by healthcare providers and policy makers to organize healthcare delivery services to reduce the treatment burden of multimorbidity, reduce healthcare costs, and improve patient outcomes. Community structure can be used to identify sets of co-occurring conditions that may benefit from a coordinated and multidisciplinary approach to healthcare delivery, while centrality analysis can be used to target conditions that may benefit from interventions aimed at prevention.

Measuring disease association, or co-occurrence, is foundational for constructing the links that form the structure of disease networks. There are several co-occurrence measures available and studies conducted to date have used a variety of different measures to construct disease networks [10, 11, 18–22]. While the intrinsic properties of co-occurrence measures have been examined [26, 27], the effect that choice of co-occurrence measure has on disease network structure and any resulting inferences has not been well studied. Substantial variation in research methods has been observed among multimorbidity studies, which challenges the comparability of research findings [28, 29]. Research comparing different methodological approaches, for studying patterns of multimorbidity, has been recommended to improve study validity and generalizability [29]. Comparing techniques for constructing multimorbidity networks could aid in determining how different techniques affect our understanding of population-level chronic disease patterns. Therefore, the purpose of this study was to assess the effect that choice of co-occurrence measure has on network analyses of co-occurring chronic conditions. Using administrative health data with excellent population coverage, separate chronic disease networks were constructed using seven co-occurrence measures. Descriptive methods were used to compare networks in terms of node centrality, community structure, and density; network edges were compared using categorized prevalence of co-occurring disease pairs.

## Methods

This retrospective cohort study was conducted using four fiscal years (April 1, 2015 – March 31, 2019) of de-identified administrative health data from the Manitoba Population Research Data Repository at the Manitoba Centre for Health Policy in the province of Manitoba, Canada. Manitoba has a universal healthcare system, therefore almost all contacts with the healthcare system for the entire population are captured in administrative health data. The provincial population is approximately 1.3 million according to the most recently-available Statistics Canada Census data.

### Data sources

Study data sources included the Manitoba Health Services Insurance Plan Registry (Population Registry), the Hospital Abstracts Database, and the Medical Services Database. These data sources were linked using a unique personal health identification number.

The Population Registry captures information on healthcare coverage for all insured Manitobans, and was used to determine eligibility for study inclusion. The Registry also includes demographic information used to characterize the study cohort. The Registry does not provide information about an individual’s gender, which is a sociological construct; it only provided information about biological sex. Individuals may change their biological sex designation.

Chronic condition diagnoses were obtained from the Hospital Abstracts Database, which contains information on discharges from hospitals, and the Medical Services Database, which records information on ambulatory services provided in physician offices. Diagnoses within hospital discharge abstracts are coded using ICD-10-CA (International Statistical Classification of Diseases and Related Health Problems, 10th Revision with Canadian Enhancements), while Medical Services diagnoses are coded with up to five digits using ICD-9-CM (International Statistical Classification of Diseases and Related Health Problems, 9th Revision, Clinical Modification).

### Cohort development

The study cohort included all Manitoba residents with complete or partial Manitoba Health insurance coverage during the study observation period. Individuals entered the study on April 1, 2015 or the date that coverage started, and were followed until the end of the study period or until their insurance coverage ceased (e.g., due to death, migration out of province). Based on information about biological sex recorded in the Population Registry, males with female-specific conditions and females with male-specific conditions were excluded (*n* = 15); the presence of these potential inconsistencies could suggest errors in the recording of diagnostic or demographic information, and it was not possible to verify the reasons for these potential inconsistencies. Since disease networks were formed from disease co-occurrence relationships, the network analysis was limited to individuals with diagnoses recorded for at least two chronic health conditions during the study observation period.

### Disease ascertainment

Chronic conditions were ascertained using diagnoses identified from inpatient discharge records in the Hospital Abstracts Database and from physician visit records in the Medical Services Database. Surgeries recorded in both data sources were also included. Prenatal and pregnancy-related records were excluded to minimize overstating disease co-occurrence among females.

A single diagnosis code was used to ascertain whether an individual was considered as having a specified condition in the study observation period. Individual diagnosis codes were grouped into 201 Expanded Diagnostic Clusters (EDC) and 27 higher-level Major Expanded Diagnostic Clusters (MEDC) using the Johns Hopkins Adjusted Clinical Group (ACG) System [30]. Diagnoses were loaded into the Johns Hopkins ACG System as World Health Organization (WHO) ICD-9 or ICD-10 codes. Five-digit ICD-10-CA codes from the Hospital Abstracts Database were truncated to the first four digits to improve compatibility with the Johns Hopkins System, which supports the WHO ICD system but not the Canadian revision. There were 49 unique Canadian-specific ICD-10-CA codes relevant to chronic disease status that were not captured by the Johns Hopkins System. These 49 Canadian-specific diagnosis codes were first translated to WHO ICD-10 codes for inclusion. An additional 17 Canadian-specific ICD-10-CA codes were not captured; however, they were irrelevant to disease status since they indicated location of occurrence or activity engaged in during occurrence. Chronic conditions classified as separate EDC categories based on severity or presence of complications were combined into single disease categories including asthma with or without asthmaticus, hypertension with or without complications, type 1 diabetes with or without complications, and type 2 diabetes with or without complications. As well, 25 EDC categories that were non-descriptive, or referred to non-chronic medical conditions or to the neonatal period were removed. Two categories indicating severity of malignant neoplasms, already classified elsewhere, were also excluded. Since co-occurrences with frequencies less than 15 were excluded from the association analysis to minimize statistical errors, seven EDC categories with low frequencies were excluded: heart murmur, lymphadenopathy, thrombophlebitis, tuberculosis infection, sinusitis, other inflammatory conditions of skin, and other female gynecologic conditions. After 34 EDC categories were excluded, 167 EDC categories remained for the network analysis.

### Disease co-occurrence measures

Disease co-occurrence was defined as two or more chronic health conditions recorded at any time during the four-year study observation period (April 1, 2015 – March 31, 2019) for the same individual. The four-year study period was selected because it was the available time frame in which diagnoses in the Medical Services dataset were coded to five digits; this detailed level of diagnostic coding was important for identifying distinct health conditions.

**Fig. 1:**
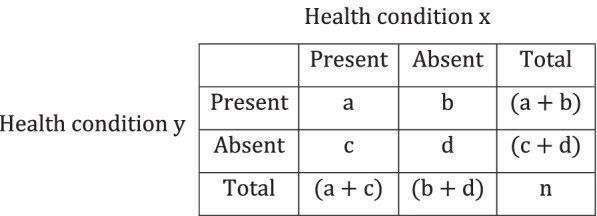
Two-way contingency table used to measure health condition associations

Disease association was measured using seven different co-occurrence measures: lift (Eq. 1), relative risk (Eq. 2), phi (Eq. 3), Jaccard (Eq. 4), cosine (Eq. 5), Kulczynski (Eq. 6), and joint prevalence (Eq. 7) [31–37]. Phi and relative risk are two of the most commonly used measures in disease network analysis, while lift is commonly used in conjunction with association rule mining. Jaccard, cosine, and Kulczynski are null-invariant measures that have been suggested for use with sparse data such as disease status datasets [26]. Joint prevalence was included due to its ease of interpretation. These measures are defined for two health conditions (i.e., x and y; see Fig. 1) as:

**Table:** 

Lift [31]	$\mathrm{n}\frac{\mathrm{a}}{\left(\mathrm{a}+\mathrm{b}\right)\left(\mathrm{a}+\mathrm{c}\right)}$	(1)
Relative risk [32]	$\frac{\mathrm{a}\left(\mathrm{c}+\mathrm{d}\right)}{\mathrm{c}\left(\mathrm{a}+\mathrm{b}\right)}$	(2)
Phi (*ϕ*) [33]	$\frac{\mathrm{ad}-\mathrm{bc}}{\sqrt{\left(\mathrm{a}+\mathrm{b}\right)\left(\mathrm{a}+\mathrm{c}\right)\left(\mathrm{b}+\mathrm{d}\right)\left(\mathrm{c}+\mathrm{d}\right)}}$	(3)
Jaccard [34]	$\frac{\mathrm{a}}{\left(\mathrm{a}+\mathrm{b}+\mathrm{c}\right)}$	(4)
Cosine [35]	$\frac{\mathrm{a}}{\sqrt{\left(\mathrm{a}+\mathrm{b}\right)\left(\mathrm{a}+\mathrm{c}\right)}}$	(5)
Kulczynski [36]	$\frac{1}{2}\left[\frac{\mathrm{a}}{\left(\mathrm{a}+\mathrm{b}\right)}+\frac{\mathrm{a}}{\left(\mathrm{a}+\mathrm{c}\right)}\right]$	(6)
Joint prevalence [37]	$\frac{\mathrm{a}}{\mathrm{n}}$	(7)

where a, b, c, d, and n are defined from the elements of a two-way contingency table.

Statistical significance of disease associations was assessed using the chi-square test when expected frequencies were greater than five, while Fisher’s exact test was used when the chi-square assumption did not hold. Associations that were not statistically significant using α = 0.01 were excluded. Since the focus of this study was on co-occurring disease, the analysis was limited to positive associations. Given that RR is an asymmetric measure of association, the maximum of the two RR measures was used.

The association analysis was limited to disease dyads. Disease association was computed using association rule mining by applying the widely-used Apriori algorithm [38]. Minimum joint frequency (called support in association rule mining) was limited to 15 to minimize statistical errors, and the minimum confidence parameter of association rule mining was left unbounded. Data preprocessing was conducted using SAS (v9.4), while R and the arules package (v1.6-7) was used to perform the association analysis [39].

### Covariates

The study cohort was characterized using age, biological sex, number of chronic conditions, residence location (urban or rural), and income quintile, an area-level measure of socioeconomic status based on average household income from the most recently-available (i.e., 2016) Statistics Canada Census [40]. Birthdate and biological sex were extracted from the most recent insurance coverage period, while socioeconomic and urban/rural status were based on the latest residence recorded during the study period. Age was calculated at exit date (i.e., the study index date) and categorized as < 20, 20-39, 40-59, 60 +.

### Network analysis

Weighted, undirected pairwise disease networks were separately constructed using each of the seven co-occurrence measures. Disease networks were stratified by the number of associations (i.e., edges) included: (a) all associations, (b) strongest 50% of associations, and (c) strongest 200 associations. Networks based on the strongest 200 associations were used to examine differences in networks that have higher visual interpretability, while networks based on the strongest 50% cut-off were used to examine how network similarity changes when a larger number of associations are included. Effect size estimates were used as edge weights and were bounded between 0 and 1 for networks measured using phi, Jaccard, cosine, and Kulczynski association measures, and unbounded for lift, relative risk, and joint prevalence.

Community structure was identified using a weighted and non-overlapping community detection algorithm developed by Blondel et al. [41]. This algorithm was chosen due to its computational efficiency on large networks and because it is widely used in applied network studies. Central nodes were identified using degree centrality and the centrality analysis was limited to the top 20 central nodes. Degree centrality was chosen because it has a clear interpretation in the context of disease co-occurrence networks (i.e., number of co-occurrence relationships) and the appropriateness of other centrality measures for use with disease co-occurrence networks is uncertain. Disease networks were visualized using the Fruchterman-Reingold force-directed layout algorithm constrained to the strongest 200 associations to improve interpretability [42]. Networks were visualized using Gephi (v0.9.2) and analyzed with Java and Gephi Toolkit (v0.9.2).

### Evaluating and comparing disease networks

Disease networks constructed using different co-occurrence measures were compared on their community structure, central nodes, common edges, network complexity, and in terms of the joint prevalence and prevalence difference distributions of their network edges.

Community structure similarity was calculated using the adjusted Rand index (ARI) with the R package aricode (v1.0.0) [43]. Community structure was also characterized by the number of communities identified and modularity, where higher modularity values indicate more distinctive communities. Important nodes, identified using degree centrality, were compared across networks by calculating their agreement, as a percent, on the top 20 central nodes. Edge similarity was calculated using the percent of edges in common between network pairs. Across all networks, overall similarity of community structure, and central node and edge agreement, was quantified using the median and the 25th (Q1) and 75th (Q3) percentiles.

Network edges were compared using categorized prevalence of co-occurring disease pairs. Based on the prevalence distribution across all 167 Johns Hopkins disease categories, disease prevalence was categorized as low (< 1%), moderate (1 to < 7%), and high (≥7%). The percent of edges in each category was used to describe the tendencies of the co-occurrence measures in estimating association strength. Joint prevalence and prevalence difference distributions were described using the median and Q1-Q3 range.

Network complexity was characterized using density (i.e., the ratio of the number of edges present in a network to the number of possible edges between all node pairs), and frequencies of nodes and edges.

## Results

### Cohort

**Fig. 2:**
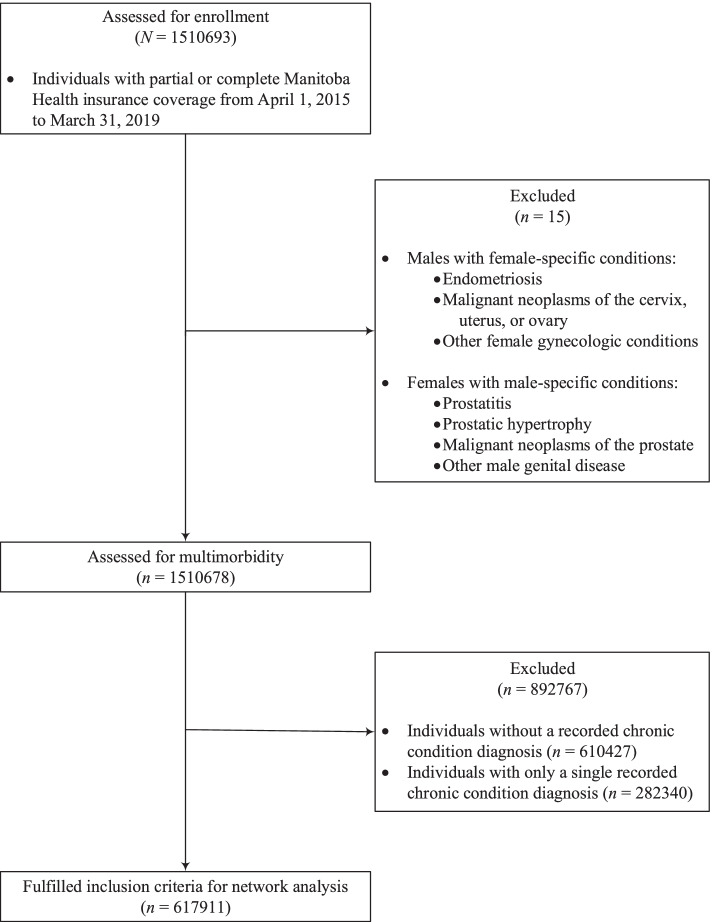
Study flow diagram

Out of 1,510,678 Manitoba residents with Manitoba Health insurance coverage between fiscal years 2015/16 and 2018/19, 610,427 (40.4%) had no chronic disease diagnosis recorded, 282,340 (18.7%) recorded a single chronic condition diagnosis, and 617,911 (40.9%) had two or more chronic condition diagnoses and were included in the network analysis (Table 1, Fig. 2). The median age of individuals with multimorbidity was considerably higher (57 years, Q1-Q3: 41-70) than individuals with one chronic condition (33 years, Q1-Q3: 18-49) or without any chronic disease (24, Q1-Q3: 11-37). There were a higher percentage of females (54.1%) and urban residents (64.1%) with multimorbidity than without (47.1% female, 61.3% urban). There were only minor differences in the distribution of socioeconomic status (income quintile) between those with and without multimorbidity.

**Table 1:** Characteristics of Manitoba residents with multimorbidity (*n* = 617,911), 2015/16-2018/19

Characteristic	N (%)
Sex	
Male	283,674 (45.9)
Female	334,237 (54.1)
Age group (years)	
< 20	43,072 (7.0)
20-39	102,750 (16.6)
49-59	189,300 (30.6)
60+	282,789 (45.8)
Residence location	
Rural	221,923 (35.9)
Urban	395,907 (64.1)
Unknown	81 (< 0.1)
Income quintile	
Q1 (lowest)	120,654 (19.5)
Q2	121,899 (19.7)
Q3	127,697 (20.7)
Q4	119,901 (19.4)
Q5 (highest)	115,384 (18.7)
Unknown	12,376 (2.0)
Chronic conditions	
2-3	304,084 (49.2)
4-5	150,938 (24.4)
6+	162,889 (26.4)

Characteristics were measured at study exit date

The five most prevalent MEDC categories in the study cohort were cardiovascular (29.1%), psychosocial (17.0%), endocrine (17.0%), musculoskeletal (12.7%), and allergy (9.4%). Hypertension was the most prevalent EDC category (22.5%), followed by depression (11.1%), disorders of lipid metabolism (9.8%), degenerative joint disease (9.1%), type 2 diabetes mellitus (9.0%), and asthma (9.0%). Hypertension was the most prevalent EDC category among both males (22.2%) and females (22.9%). Following hypertension, the most prevalent EDC categories among males were disorders of lipid metabolism (10.5%), type 2 diabetes (9.4%), asthma (8.2%), depression (7.7%), and degenerative joint disease (7.5%); while depression (14.4%), degenerative joint disease (10.7%), asthma (9.9%), disorders of lipid metabolism (9.1%), and hypothyroidism (8.9%) were the next most prevalent conditions among females.

### Disease association analysis

Out of 9138 pairwise disease co-occurrences with joint frequencies ≥15, 1293 (14.1%) were excluded due to being either non-significant (i.e., *p*-value > 0.01) or non-positive (i.e., phi < 0). After exclusions, 7845 associations remained for the analysis. Since network density is not modified by edge weight, network density was constant (0.57) for all seven networks constructed with different co-occurrence measures when all edges (*n* = 7845) were included (N nodes = 166).

Networks constructed by limiting the number of associations differed in their density and number of nodes (Table 2). For pairwise networks constructed using the strongest 200 associations, the network with the least number of nodes (*n* = 56 for joint prevalence) had the highest network density (0.13), while the two networks with the greatest number of nodes (*n* = 114 for relative risk*; n* = 123 for Kulczynski) had the lowest network density at 0.03. As more associations were included, variation in the number of nodes and network density decreased between the networks. For the pairwise networks constructed with the strongest 50% of associations (i.e., *n* = 3922 associations), the number of nodes ranged from 150 to 166 and network density varied between 0.29 and 0.35.

**Table 2:** Global properties for multimorbidity networks constructed with select association measures

Association measure	Top 200 associations^a^				Top 50% of associations^b^			
	N nodes	Density	Modularity	N communities	N nodes	Density	Modularity	N communities
Lift	108	0.04	0.72	13	165	0.29	0.30	6
Relative risk	114	0.03	0.60	13	166	0.29	0.43	5
Phi	87	0.05	0.43	17	164	0.29	0.19	4
Jaccard	72	0.08	0.37	14	150	0.35	0.16	5
Cosine	73	0.08	0.37	11	161	0.31	0.15	4
Kulczynski	123	0.03	0.37	8	166	0.29	0.14	5
Joint prevalence	56	0.13	0.08	3	151	0.35	0.07	2

^a^Disease network was limited to the 200 strongest associations (i.e., largest effect size)

^b^Disease network was limited to the strongest 50% (*n* = 3922) of all statistically significant associations

### Network visualization

**Fig. 3:**
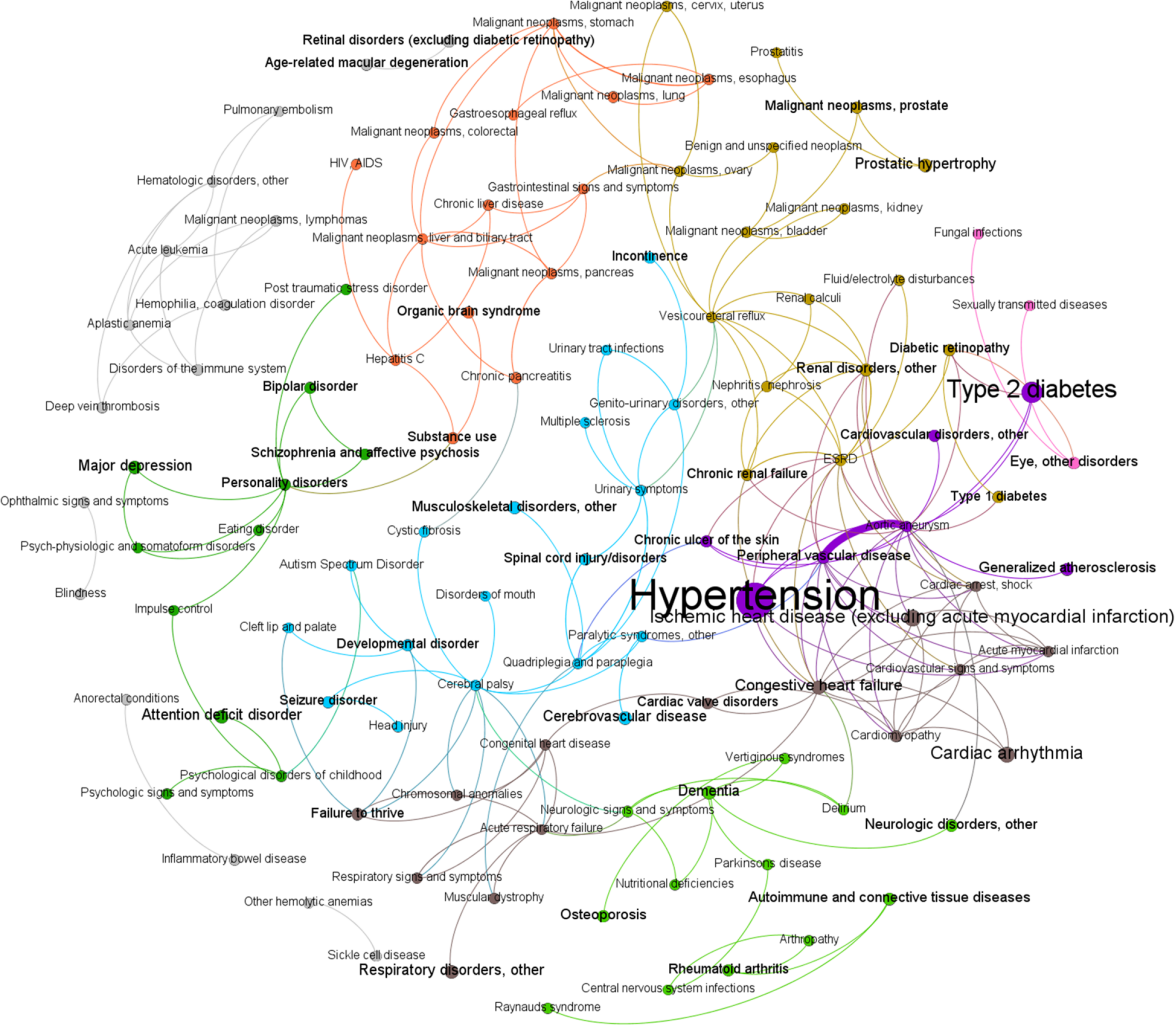
Multimorbidity network with associations measured using relative risk, limited to the 200 strongest associations. Node diameter and font size are proportional to prevalence, edge weight (thickness) is proportional to effect size, and node and edge color indicate community structure (i.e., disease clusters). ESRD = end-stage renal disease, HIV/AIDS = human immunodeficiency virus/acquired immunodeficiency syndrome

**Fig. 4:**
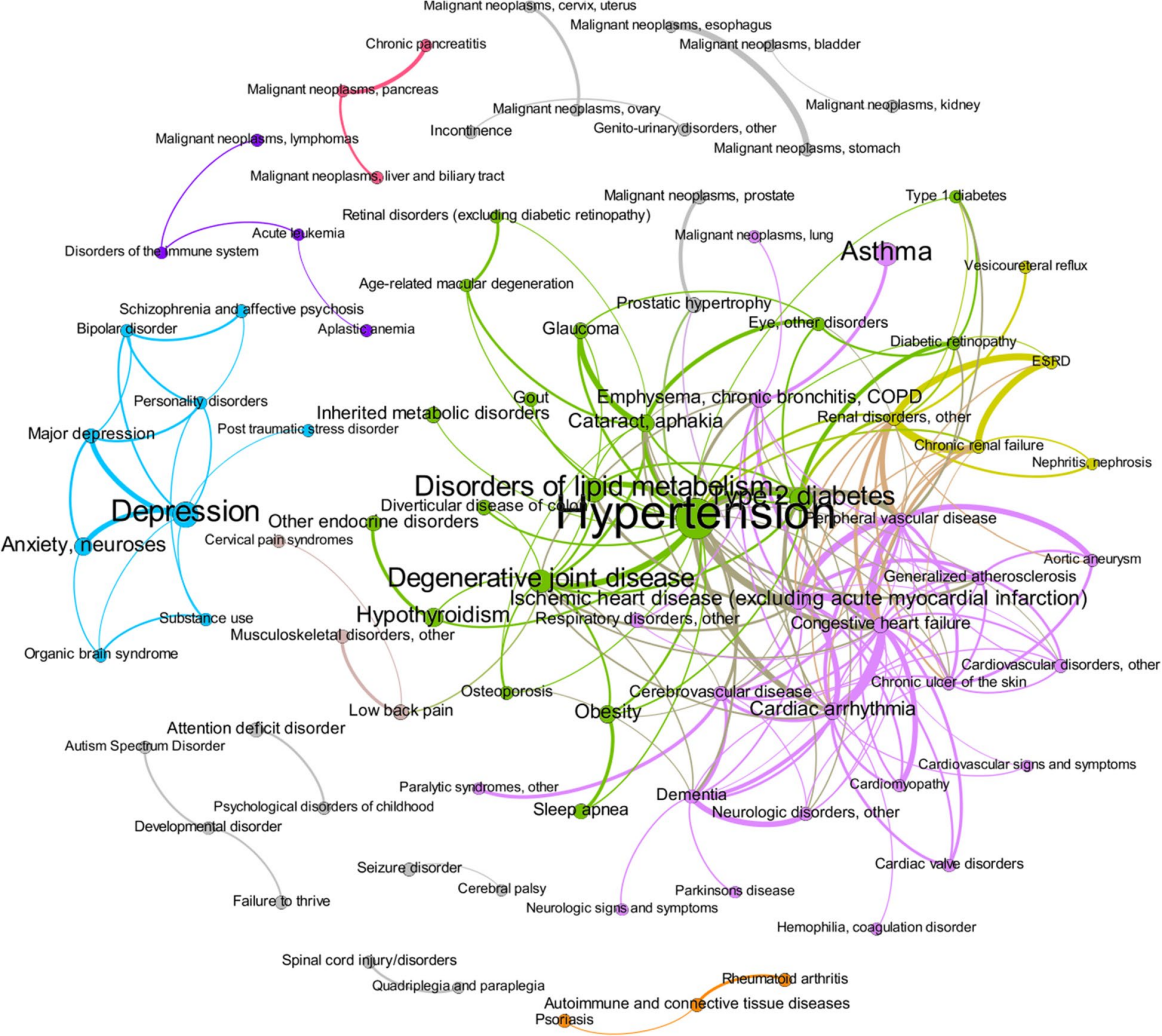
Multimorbidity network with associations measured using phi, limited to the 200 strongest associations. Node diameter and font size are proportional to prevalence, edge weight (thickness) is proportional to effect size, and node and edge color indicate community structure (i.e., disease clusters). COPD = chronic obstructive pulmonary disease, ESRD = end-stage renal disease

Including all statistically significant pairwise associations produced dense network visualizations that were difficult to interpret (Supplementary Fig 1, Additional File 1). Reducing complexity by selecting the strongest 200 EDC associations produced more interpretable network diagrams (Figs. 3 & 4; Supplementary Figs. 2-6, Additional File 1). Visual interpretability of the disease networks limited to the top 200 associations varied depending on the association measure used to construct the network.

### Co-occurrence relationships characterized by disease prevalence

**Fig. 5:**
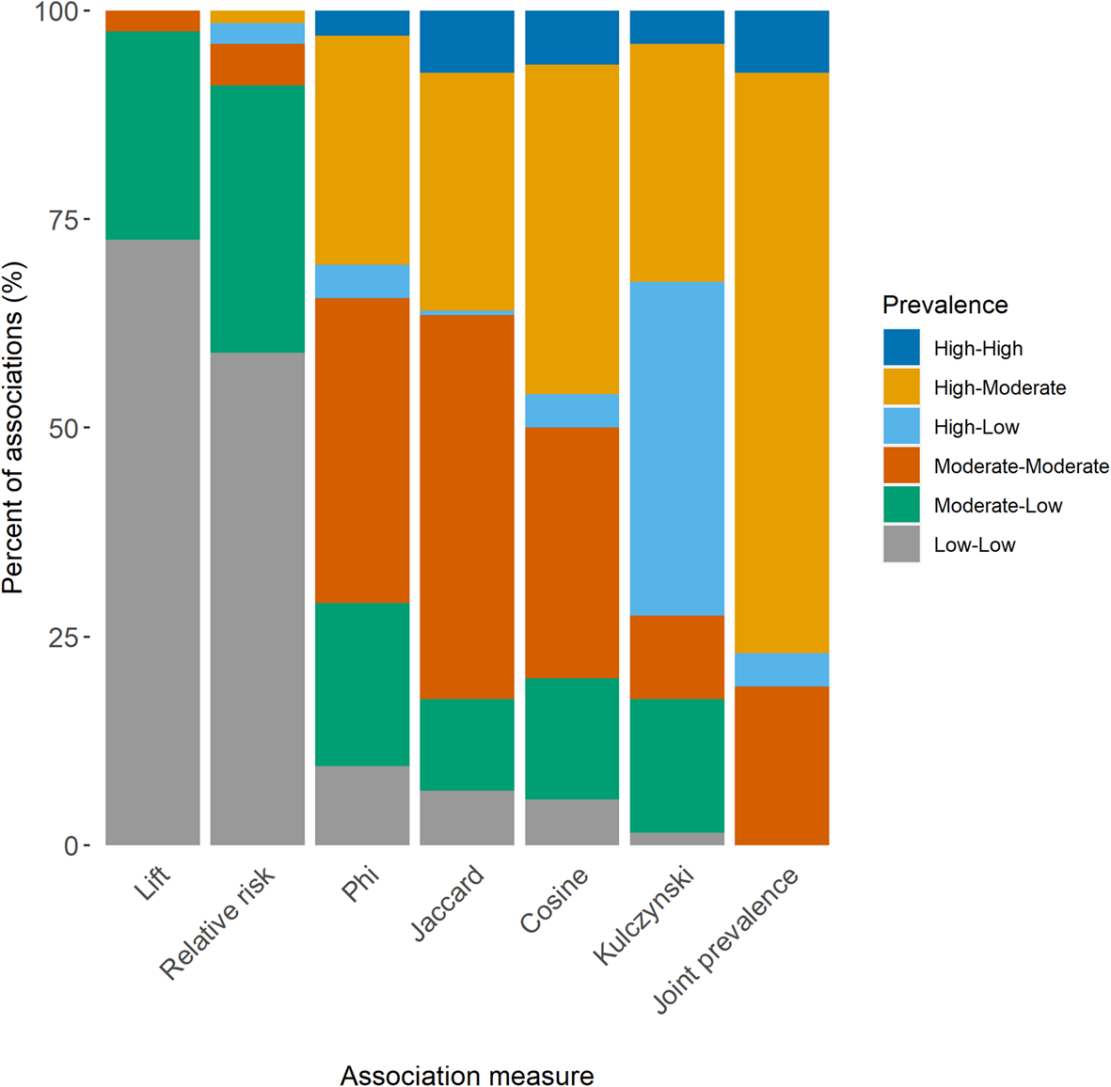
Percent of the 200 strongest associations characterized by prevalence, among select association measures. Prevalence was categorized as low (< 1%), moderate (1 to < 7%), and high (≥7%)

Different co-occurrence measures estimate higher association strengths for different types of relationships, in terms of the prevalence difference between disease pairs. These preferences by association measures result in certain pairwise chronic disease relationships being emphasized more than other combinations, when limiting networks to the strongest associations. Differences based on disease prevalence were more pronounced between networks when using a smaller number of the strongest associations and decreased when including 50% (*n* = 3922) of all statistically significant associations (Table 3, Fig. 5; Supplementary Figs. 7 & 8, Additional File 1).

**Table 3:** Joint prevalence and prevalence difference distributions among multimorbidity networks constructed with select association measures

Association measure	Top 200 associations^a^		Top 50% of associations^b^	
	Joint prevalence	Prevalence difference	Joint prevalence	Prevalence difference
Lift	0.0 (0.0-0.0)	0.3 (0.1-0.8)	0.0 (0.0-0.0)	0.9 (0.4-1.9)
Relative risk	0.0 (0.0-0.1)	0.4 (0.1-1.3)	0.0 (0.0-0.0)	1.0 (0.4-2.1)
Phi	0.4 (0.2-0.9)	2.2 (0.6-6.3)	0.0 (0.0-0.1)	1.4 (0.6-3.4)
Jaccard	0.5 (0.3-1.1)	2.0 (0.5-5.0)	0.0 (0.0-0.1)	1.1 (0.4-2.4)
Cosine	0.6 (0.3-1.2)	3.2 (1.1-7.7)	0.0 (0.0-0.1)	1.5 (0.6-3.8)
Kulczynski	0.4 (0.1-0.9)	17.9 (3.6-22.0)	0.0 (0.0-0.1)	2.3 (1.1-4.7)
Joint prevalence	0.7 (0.6-1.2)	6.8 (3.4-12.9)	0.0 (0.0-0.1)	1.7 (0.7-4.1)

Data are presented as median (Q1-Q3), where Q1 = 25th percentile and Q3 = 75th percentile

Joint prevalence and prevalence difference measured between disease node pairs

^a^Disease network was limited to the 200 strongest associations (i.e., largest effect size)

^b^Disease network was limited to the strongest 50% (*n* = 3922) of all statistically significant associations

Networks based on lift and relative risk accentuated co-occurrence relationships between pairs of low prevalent (< 1%) conditions, at 72.5 and 59.0% respectively (Table 3, Fig. 5). The percentage of edges highlighting co-occurrences between two low prevalent conditions in the other five networks ranged from 0% (joint prevalence) to 9.5% (phi). Lift and relative risk also highlighted a higher proportion of relationships between moderately prevalent (1 to < 7%) and low prevalent conditions, compared with the other co-occurrence measures.

Relationships between two moderately prevalent conditions were emphasized by phi, Jaccard, and cosine based networks: 36.5, 46.0, and 30.0%, respectively. Phi, Jaccard, and cosine also emphasized relationships between highly and moderately prevalent diseases: 27.5, 28.5, and 39.5%, respectively. The majority of the edges in the Kulczynski-based network represented relationships between conditions of high and low prevalence (40.0%), and between highly prevalent and moderately prevalent conditions (28.5%). Relationships between conditions of high and low prevalence only constituted up to 4.0% of all edges in the other six networks.

Measuring co-occurrence using joint prevalence resulted in the highest percentage of edges connecting highly prevalent and moderately prevalent disease nodes (69.5%). Joint prevalence and Jaccard produced networks with the most connections between two highly prevalent conditions (7.5%). Correspondingly, the joint prevalence network had the highest median joint prevalence (0.7%, Q1-Q3: 0.6-1.2%). Lift and relative risk based networks did not contain any edges between two highly prevalent disease nodes, while associations between pairs of highly prevalent conditions accounted for 3.0 to 6.5% of the edges in networks built using phi, cosine, and Kulczynski.

The median difference in prevalence between pairs of co-occurring conditions was lowest for lift (0.3%, Q1-Q3: 0.1-0.8%) and relative risk (0.4%, Q1-Q3: 0.1-1.3%); and highest for Kulczynski (17.9%, Q1-Q3: 3.6-22.0%) (Table 3). There was less variation in the distribution of prevalence differences among the seven co-occurrence measures when 50% of all statistically significant associations were included.

### Network edge similarity

**Fig. 6:**
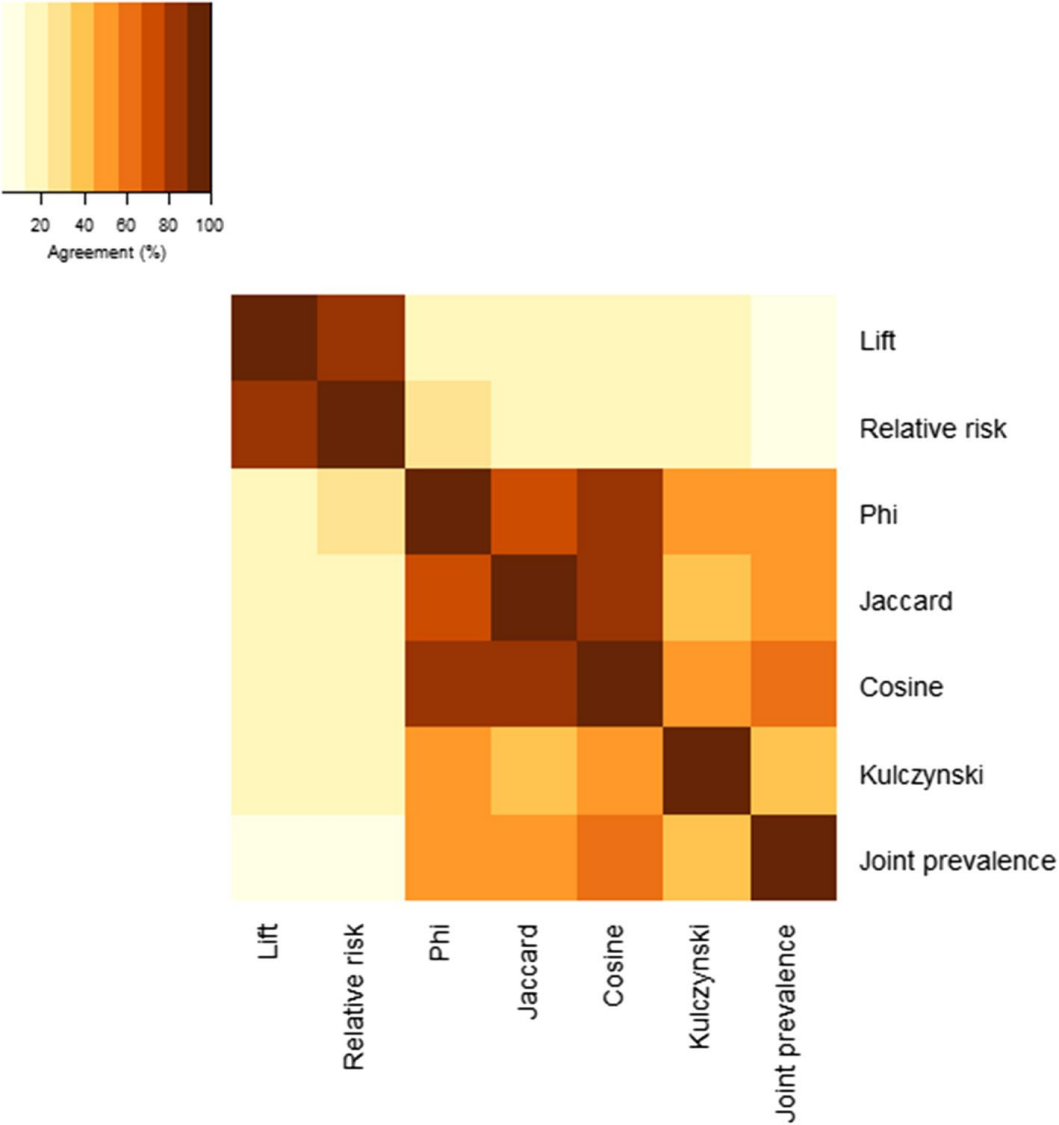
Percent of the 200 strongest associations in common between networks constructed using different association measures

Disease networks constructed using different co-occurrence measures were dissimilar in terms of the edges included in the top 200 associations (Fig. 6), with edge agreement (percentage of co-occurrence relationships in common) ranging from 1.5% for lift and joint prevalence to 86.5% for lift and relative risk. Phi- and Jaccard-based networks had moderate agreement with the cosine-based network (83.0 and 79.5%). Phi and Jaccard had moderate agreement (78.0%), while the remaining network pairs had lower agreement with a range from 5.0 to 63.5%. Median agreement (37.0%, Q1-Q3: 20.0-53.5%) among the network pairs was much lower when limited to the 200 strongest associations, than when the top 50% of all statistically significant associations were used to construct the networks (68.5%, Q1-Q3: 58.7-83.9%; Supplementary Fig. 11, Additional File 1).

### Community structure

Community structure differed considerably amongst networks constructed using different co-occurrence measures. The number of communities detected had the largest range (3 to 17) between networks limited to 200 strongest associations (Table 2). However, networks also had considerable dissimilarity in the number of communities detected when all statistically significant associations were included (3 to 7) and when limited to the strongest 50% of all statistically significant associations (2 to 6). Modularity, a measure of how well a network separates into communities, also widely varied between networks limited to the 200 strongest associations (0.08 to 0.72) but variation decreased when networks included the top 50% of all significant associations (0.07 to 0.43). When all statistically significant associations were included, modularity ranged from 0.07 (joint prevalence) to 0.36 (relative risk).

**Fig. 7:**
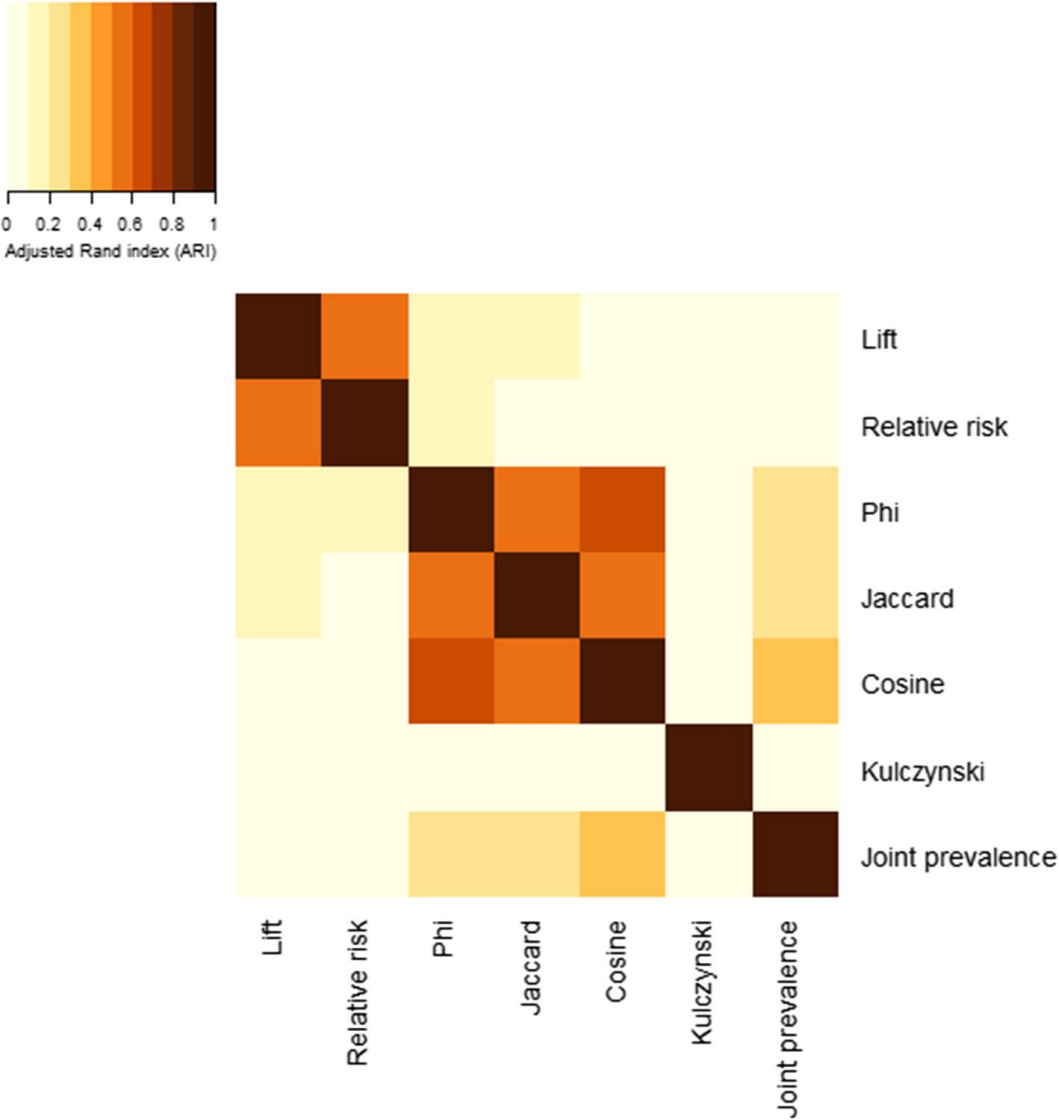
Community structure similarity between multimorbidity networks limited to the 200 strongest associations. Community structure similarity was measured using the adjusted Rand index (ARI)

Community structure similarity, as measured using the ARI, was strongest between phi and cosine in networks limited to the top 200 associations (ARI = 0.68) (Fig. 7), and between relative risk and lift (ARI = 0.49) and phi and cosine (ARI = 0.48) among networks limited to the top 50% of all significant associations (Supplementary Fig. 12, Additional File 1). Overall, co-occurrence measurement differences resulted in poor community structure similarity: the median ARI was 0.08 (Q1-Q3: 0.06-0.24) for networks including the top 200 associations and the median ARI was 0.26 (Q1-Q3: 0.24-0.32) for networks consisting of the top 50% of associations.

### Nodes of importance

Since degree centrality is a non-weighted measure, networks that included all statistically significant edges without limiting inclusion by effect size had identical degree distributions. When including all statistically significant associations, the five chronic condition categories with the highest degree centrality were: other endocrine disorders, depression, major depression, sleep apnea, and asthma.

**Fig. 8:**
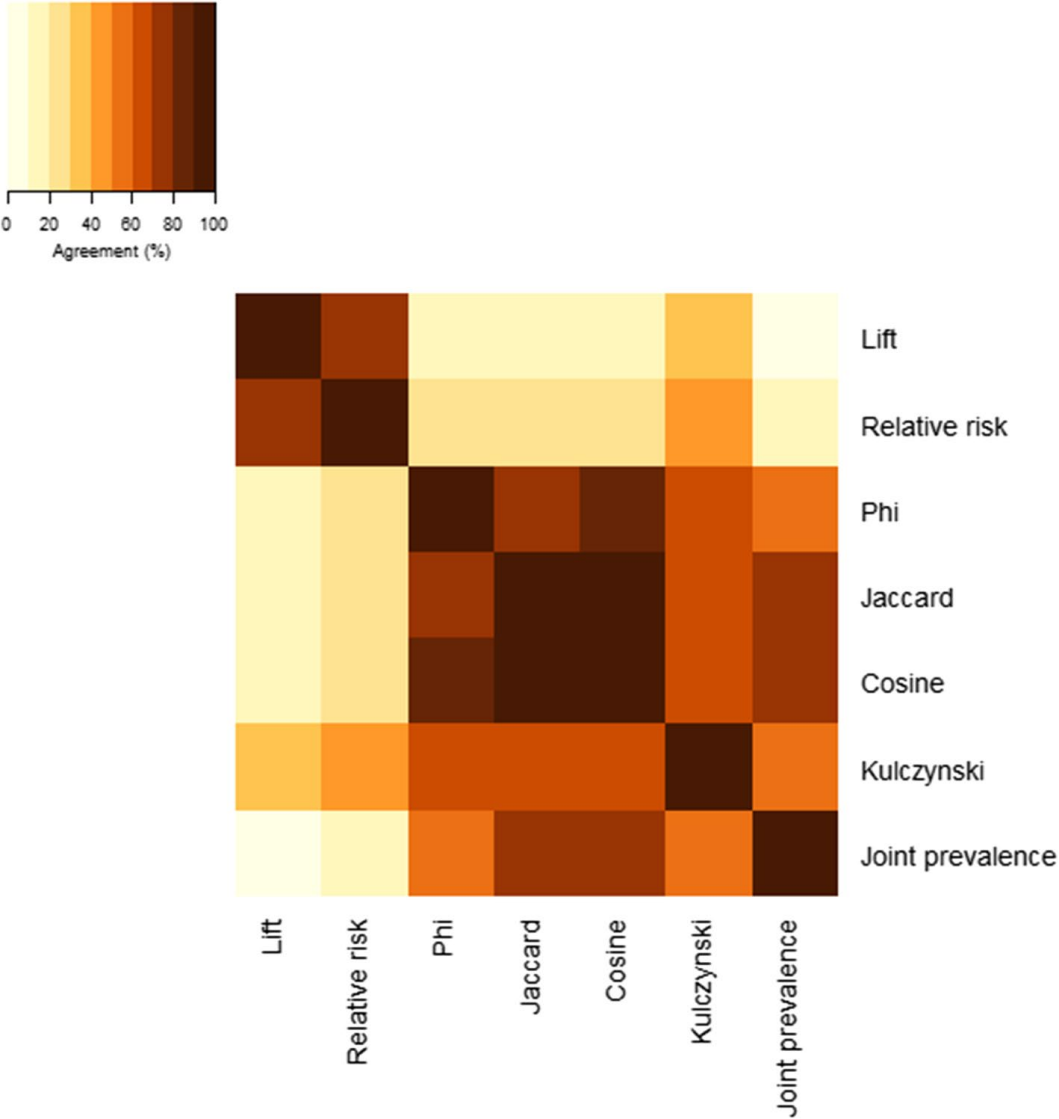
Agreement on the 20 most central nodes between networks limited to the 200 strongest associations. Node centrality was measured using degree centrality

The selection of the top 20 disease categories with the highest degree centrality varied amongst networks constructed using different co-occurrence measures. Networks had a median agreement of 55.0% (Q1-Q3: 25.0-75.0%) when limited to the top 200 co-occurrence relationships (Fig. 8; Supplementary Table 1, Additional File 1) and a median agreement of 55.0% (Q1-Q3: 30.0-75.0%) when limited to the strongest 50% of all significant associations (Supplementary Fig. 13, Additional File 1). When limited to the top 200 co-occurrences, agreement ranged from 5% between lift and joint prevalence to 95% between Jaccard and cosine. Agreement between two of the most commonly used measures among disease network studies, relative risk and phi, agreed on only 30% of the top 20 central nodes. When 50% of all statistically significant associations were included, agreement was strongest between Kulczynski and joint prevalence (95% agreement), and weakest between lift and Kulczynski (20%) and between lift and joint prevalence (20%).

## Discussion

Measuring disease co-occurrence is essential when constructing multimorbidity networks to determine the connecting links between disease nodes and the strengths of these co-occurrence relationships, which serve as edge weights. Different association measures highlight different co-occurrence relationships, in terms of disease prevalence, based on which relationships are assigned higher association estimates. In weighted disease networks where effect size estimates are used as edge weights, differences in co-occurrence measurement influence community detection algorithms and node centrality measures that use edge weights in their calculations. Unweighted measures such as network density and degree centrality are not affected by the choice of co-occurrence measures unless network links are excluded based on effect size cut-offs. When limiting the number of edges in a network by effect size, to produce a visually interpretable diagram, choice of co-occurrence measure can have a significant impact on network structure and network analysis inferences. Evaluating the accuracy or validity of a network requires a ground truth against which to compare network structure. Since there is no ground truth for a chronic disease co-occurrence network, this study used descriptive methods to highlight the impact that co-occurrence measurement has on network analysis.

This study showed the majority of the highest associations measured using lift and relative risk pertained to co-occurrence relationships between pairs of low prevalence conditions. In contrast, the strongest associations in the joint prevalence network included highly-prevalent conditions, while the Kulczynski measure emphasized relationships between high and low prevalence conditions. Phi, Jaccard, and cosine emphasized associations with moderately-prevalent conditions. Comparing Jaccard and cosine, Jaccard tended to prefer co-occurrence relationships between diseases of similar prevalence, while cosine assigned slightly less emphasis to events of similar frequency.

The results from the current study concur with Hidalgo et al., who compared disease co-occurrence networks constructed using RR and *ϕ* and found the network constructed with RR to have a greater number of low prevalence conditions and the *ϕ*-based network to be characterized by more prevalent conditions [10]. In addition to describing network edges by disease prevalence, the current study also showed the impact that disease co-occurrence measurement has on community structure and node centrality—items not discussed previously in literature. Along with contrasting RR and *ϕ*, this study also compared disease networks constructed using lift, a measure commonly used in conjunction with association rule mining, and null-invariant measures (cosine, Jaccard, and Kulczynski), which have been suggested for use with sparse data such as disease status matrices [26]. The differences amongst the null-invariant measures observed in the current study agree with Wu et al., who described the preference of Jaccard for relationships between events of similar frequency, Kulczynski for relationships between frequent and rare events, and cosine as being situated between these two in terms of the relationships that receive the highest association estimates [27].

Although there has been only limited research about the effect that the choice of association measure has on disease network structure, some studies have examined the intrinsic properties of association measures including the inversion invariance and null invariance properties, which are important considerations when choosing an appropriate measure of association [26, 27]. The inversion invariance property refers to stable association estimates when events (i.e., presence of disease) and non-events (i.e., values indicating absence of disease) are flipped [26]. Association measures that are inversion invariant, such as *ϕ*, assign equal importance to co-presence and co-absence and their association estimates remain constant when disease status is inversed. Association measures that are not inversion invariant may be better suited for asymmetric binary data, such as disease co-occurrence data, where absence of disease outweighs the number of positive cases [26]. Null invariance refers to constant effect estimates when there is an increase in the total number of records with neither event of interest (i.e., an increase in the number of disease-free individuals). Cosine, Jaccard, and Kulczynski are null-invariant measures of association, while *ϕ* violates the null invariance property. Lift, RR, and joint prevalence are neither inversion invariant nor null invariant. Like inversion invariance, null invariance is an important consideration for disease co-occurrence analysis since disease status matrices typically contain a large proportion of null transactions (observations that do not contain any of the events of interest) [27]. However, assessing the appropriateness of an association measure is still difficult even after an examination of its intrinsic properties. By outlining the tendencies of association measures to emphasize certain types of co-occurrence relationships, our study provides an additional empirical basis to aid researchers in selecting an appropriate co-occurrence measure.

The current study has a number of strengths. Extracting diagnoses from both hospital and physician data aids in providing a comprehensive picture of chronic disease patterns in the Manitoba population [44]. Furthermore, the administrative health data used in this study had excellent population coverage since the data are based on a single public insurer that effectively captures healthcare system encounters for all Manitoba residents, with few exceptions—resulting in excellent generalizability of the observed chronic disease patterns at the population level. Utilizing precise ICD diagnostic codes (i.e., up to five digits) minimized misclassification errors and allowed for the definition of certain disease categories that cannot be distinguished from one another when only using 3-digit codes. Finally, the large number of chronic condition categories under analysis facilitated the examination of many potentially interesting disease patterns that are obscured when using a more limited number of categories based on a comorbidity index.

Despite the strengths of this study, there are some limitations. The true distribution of chronic disease in the underlying population can differ significantly from disease patterns observed within administrative claims data. Since chronic conditions were defined through contact with the healthcare system, disease information may have been inadequately captured for individuals with limited access to healthcare services or for conditions which individuals are less likely to seek treatment. Consequently, there will be missing links or underestimated edge weights for relationships involving underreported health conditions within the structure of the disease co-occurrence networks. To increase diagnostic precision, this study was constrained to the 4-year period when diagnoses in physician billing claims were coded with up to five digits; but in doing so this study did not capture diagnoses that were only recorded in earlier time periods. This reduced observation period may have resulted in understating co-occurrence for less prevalent conditions or conditions that are infrequently documented in billing claims. All diagnoses observed during the 4-year study period for a specific individual were treated as persisting during the entire time period, which may have resulted in overstating certain co-occurrence relationships since diseases that may have been in remission were still considered as co-occurring with other conditions after the point of remission. Due to the relatively large number of chronic condition categories under consideration, it was not feasible to use complex case definitions to ascertain disease status based on diagnosis code counts. Simplified case definitions based on single diagnosis codes were used to mark disease status and misclassification may have occurred due to diagnostic coding errors. Finally, the constructed disease networks can be useful for generating hypotheses and visualizing disease patterns; however, disease progression was not considered in this study and network properties, such as node centrality, should not be used for causal inference [45].

Researchers must make several methodological choices when seeking to conduct a multimorbidity network analysis. In addition to choosing a measure of association, researchers must choose from many different community detection techniques, and node centrality and network complexity measures. While this study discusses approaches to choosing an association measure, researchers seeking to conduct a disease co-occurrence network analysis will also benefit from future studies that develop guidelines on choosing from these other network methods. Administrative health data are available in many jurisdictions and the methodology used in the current study can be applied to compare population-level chronic disease patterns across jurisdictions and between population sub-groups defined by determinants of health such as region of residence or socioeconomic status.

## Conclusions

The choice of co-occurrence measure affects our interpretation of population-level multimorbidity patterns obtained using network analysis by influencing which diseases co-occur with many other conditions within a population (i.e., node centrality), how disease clusters are defined (i.e., network community structure), and network complexity estimates (i.e., network density). Comparing networks constructed using different co-occurrence measures, many of the diseases that clustered together were similar but clusters differed by size and in terms of the nodes that were central to the clusters. Although the selection of a co-occurrence measure can be challenging given the lack of a ground truth to evaluate against, knowing the tendencies of different co-occurrence measures allows researchers to make informed choices based on their research goals. Co-occurrence measures should be selected considering their intrinsic properties, research objectives, and the health condition prevalence relationships of greatest interest. Researchers should consider conducting sensitivity analyses using different co-occurrence measures.

## Supplementary Information

**Additional file 1.**

## Data Availability

Data used in this article was derived from administrative health data as secondary use. The data was provided to the investigators under specific data sharing agreements only for approved use at Manitoba Centre for Health Policy (MCHP). The original source data is not owned by the researchers or MCHP and as such cannot be provided to a public repository. The original source data are available with submission of appropriate ethics approval forms to the Health Research Ethics Board of the University of Manitoba (see https://www.umanitoba.ca/research/orec/ethics_medicine/forms.html for more details), and data access approval forms to the Provincial Health Research Privacy Committee (see https://www.rithim.ca/phrpc-submission-information for more details).

## Abbreviations

ACG: Adjusted Clinical Group
AIDS: Acquired immunodeficiency syndrome
ARI: Adjusted Rand index
COPD: Chronic obstructive pulmonary disease
EDC: Expanded Diagnostic Cluster
ESRD: End-stage renal disease
HIV: Human immunodeficiency virus
ICD: International Statistical Classification of Diseases and Related Health Problems
ICD-10-CA: International Statistical Classification of Diseases and Related Health Problems, 10th Revision, Canada
ICD-9-CM: International Statistical Classification of Diseases and Related Health Problems, 9th Revision, Clinical Modification
MEDC: Major Expanded Diagnostic Cluster
RR: Relative risk
WHO: World Health Organization
