# Supplementary material for: The effect of disease co-occurrence measurement on multimorbidity networks: a population-based study
Source: BMC Med Res Methodol. 2022 Jun 8;22:165. doi: 10.1186/s12874-022-01607-8 (PMC9175465; doi:10.1186/s12874-022-01607-8)
Supplement: Supplementary file 1 — Additional file 1. [file 12874_2022_1607_MOESM1_ESM.docx]

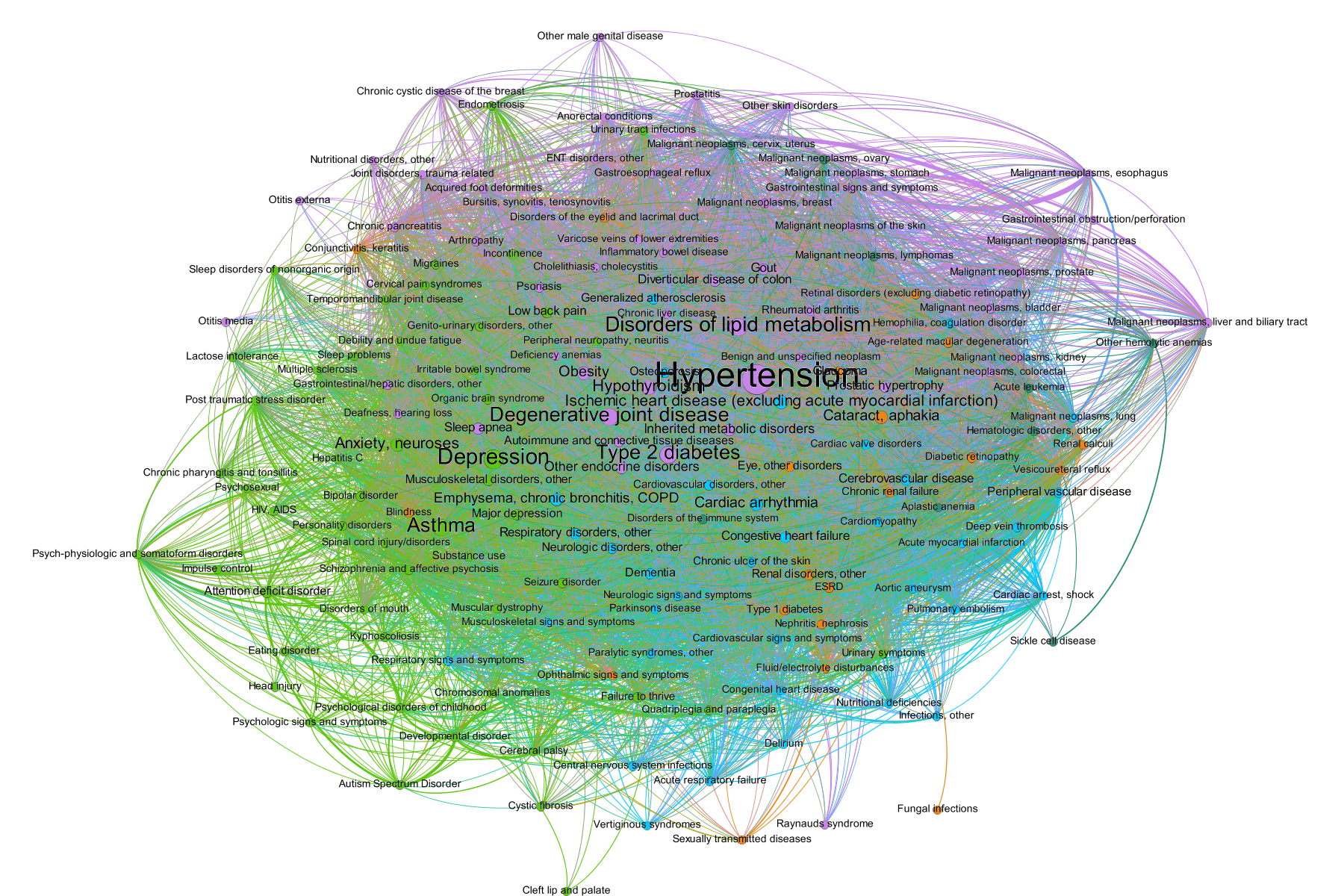


Supplementary Figure 1. Multimorbidity network constructed using all statistically significant associations (*n*=7845), with associations measured using phi

Node diameter and font size are proportional to prevalence, edge weight (thickness) is proportional to effect size, and node and edge color indicate community structure (i.e., disease clusters). COPD = chronic obstructive pulmonary disease; ENT = ear, nose, and throat; ESRD = end-stage renal disease; HIV/AIDS = human immunodeficiency virus/acquired immunodeficiency syndrome


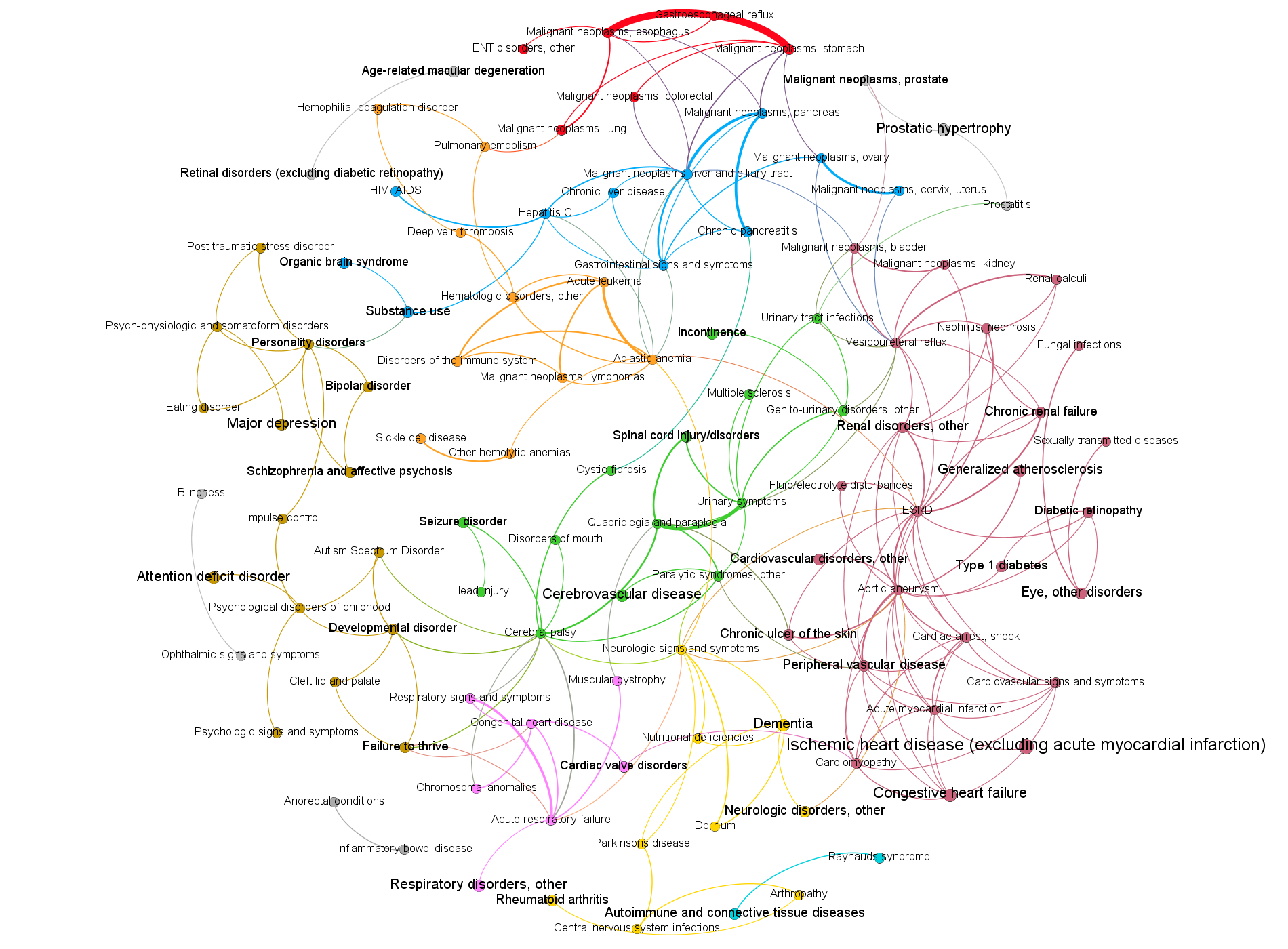


Node diameter and font size are proportional to prevalence, edge weight (thickness) is proportional to effect size, and node and edge color indicate community structure (i.e., disease clusters). ENT = ear, nose, and throat; ESRD = end-stage renal disease; HIV/AIDS = human immunodeficiency virus/acquired immunodeficiency syndrome

Supplementary Figure 2. Multimorbidity network with associations measured using lift, limited to the 200 strongest associations

Supplementary Figure 3. Multimorbidity network with associations measured using Jaccard, limited to the 200 strongest associations


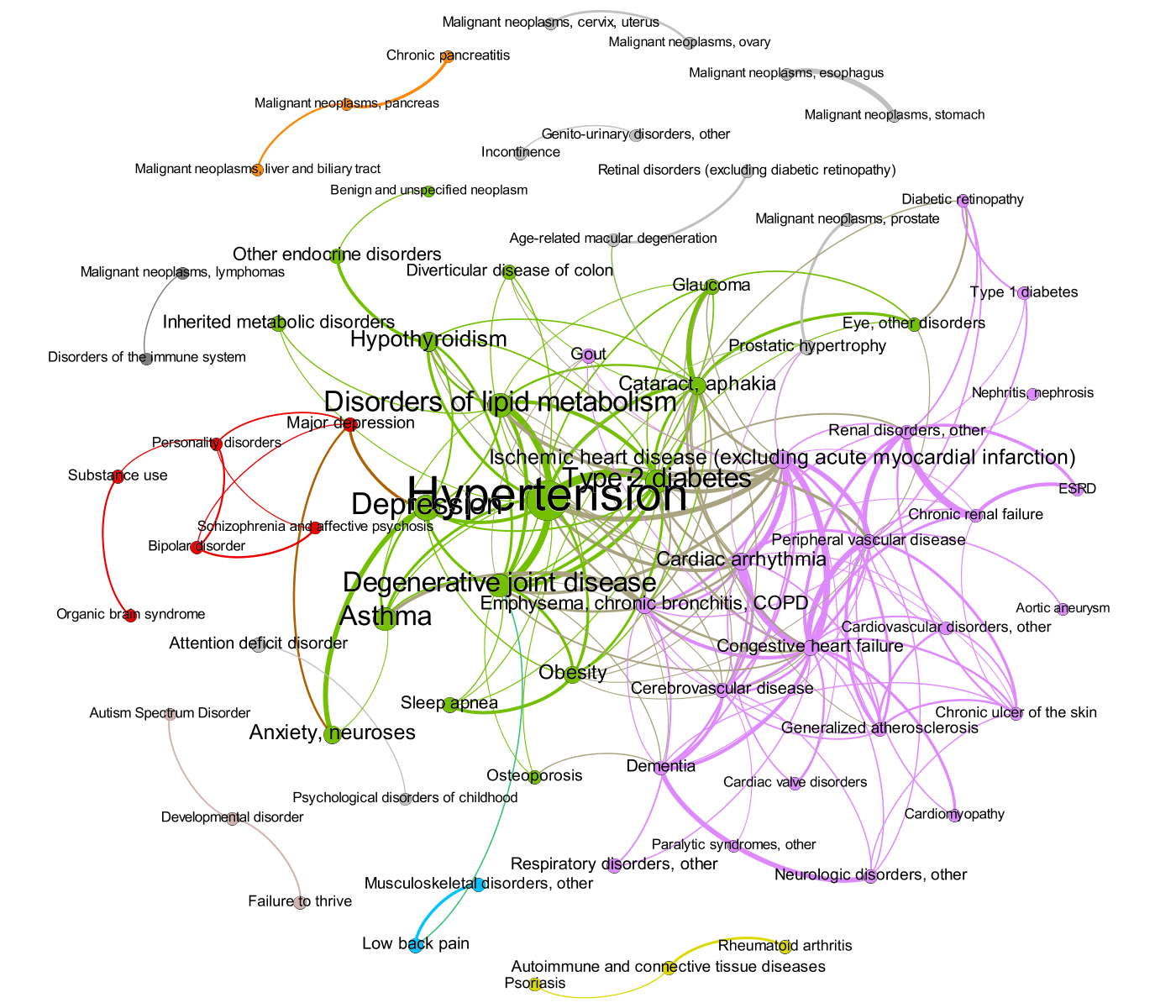


Node diameter and font size are proportional to prevalence, edge weight (thickness) is proportional to effect size, and node and edge color indicate community structure (i.e., disease clusters). COPD = chronic obstructive pulmonary disease, ESRD = end-stage renal disease


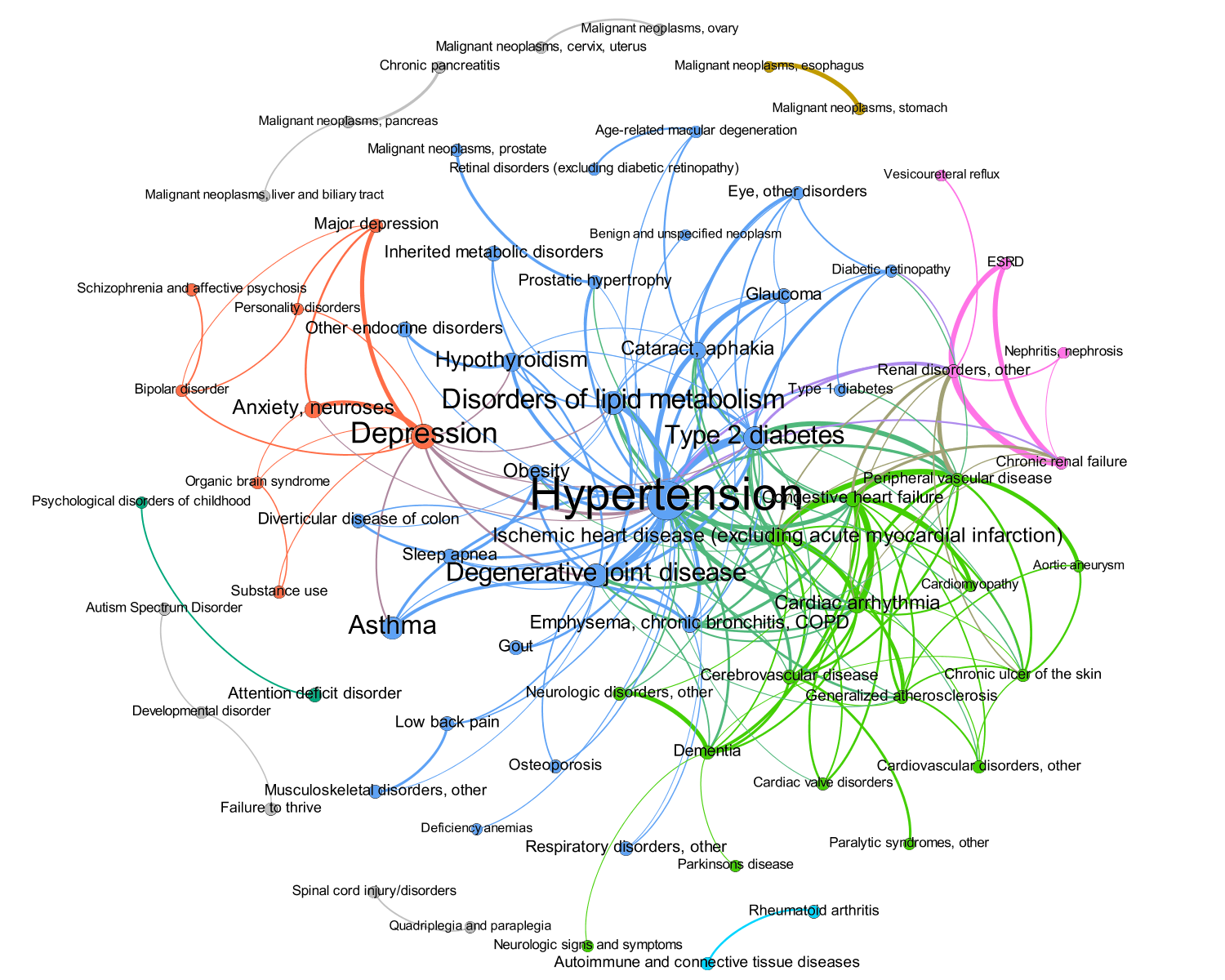


Node diameter and font size are proportional to prevalence, edge weight (thickness) is proportional to effect size, and node and edge color indicate community structure (i.e., disease clusters). COPD = chronic obstructive pulmonary disease, ESRD = end-stage renal disease

Supplementary Figure 4. Multimorbidity network with associations measured using cosine, limited to the 200 strongest associations


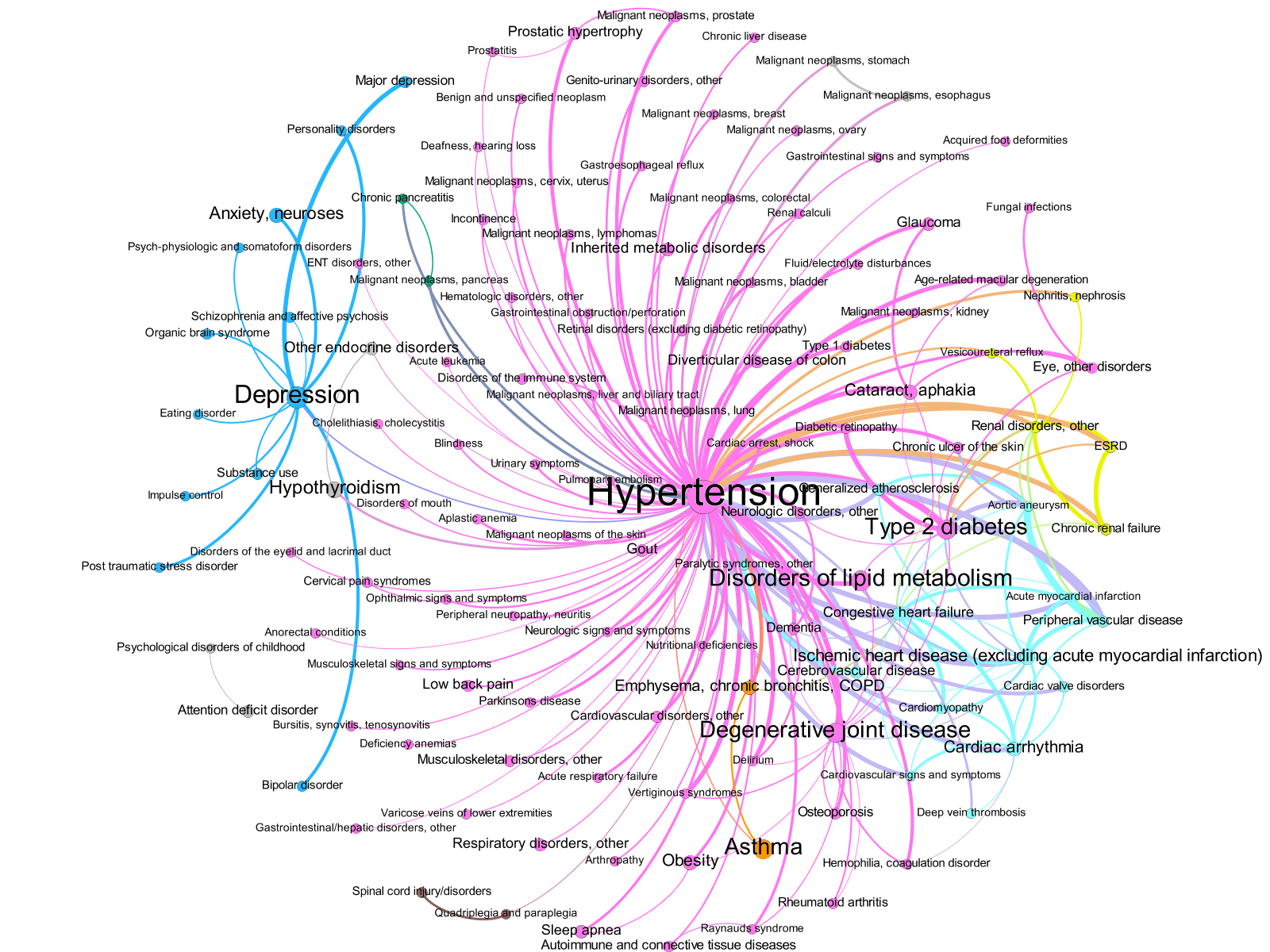


Node diameter and font size are proportional to prevalence, edge weight (thickness) is proportional to effect size, and node and edge color indicate community structure (i.e., disease clusters). COPD = chronic obstructive pulmonary disease; ENT = ear, nose, and throat; ESRD = end-stage renal disease

Supplementary Figure 5. Multimorbidity network with associations measured using Kulczynski, limited to the 200 strongest associations


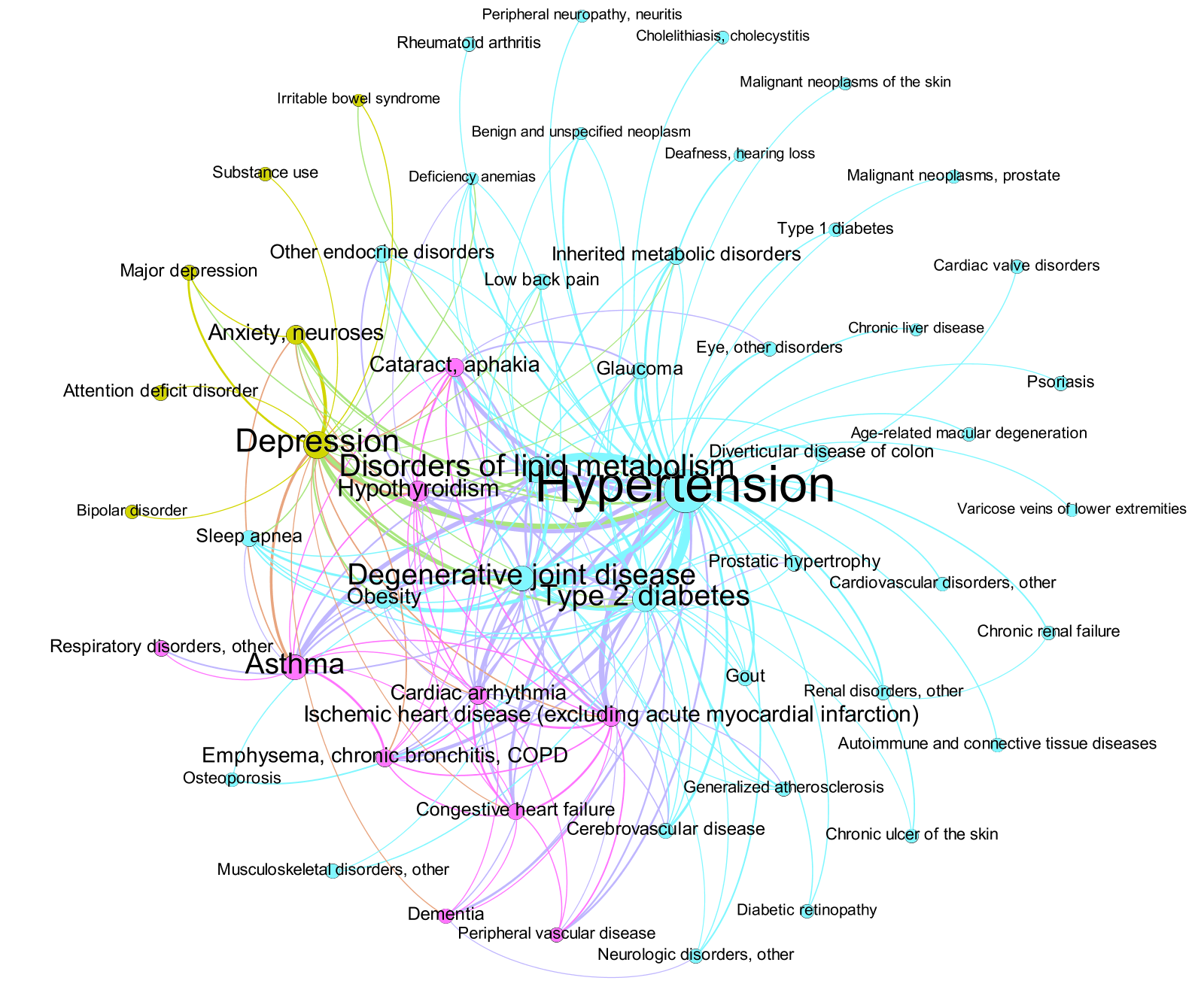


Node diameter and font size are proportional to prevalence, edge weight (thickness) is proportional to effect size, and node and edge color indicate community structure (i.e., disease clusters). COPD = chronic obstructive pulmonary disease

Supplementary Figure 6. Multimorbidity network with associations measured using joint prevalence, limited to the 200 strongest associations

Supplementary Figure 7. Percent of the strongest 50 percent (*n*=3922) of all statistically significant associations, characterized by prevalence


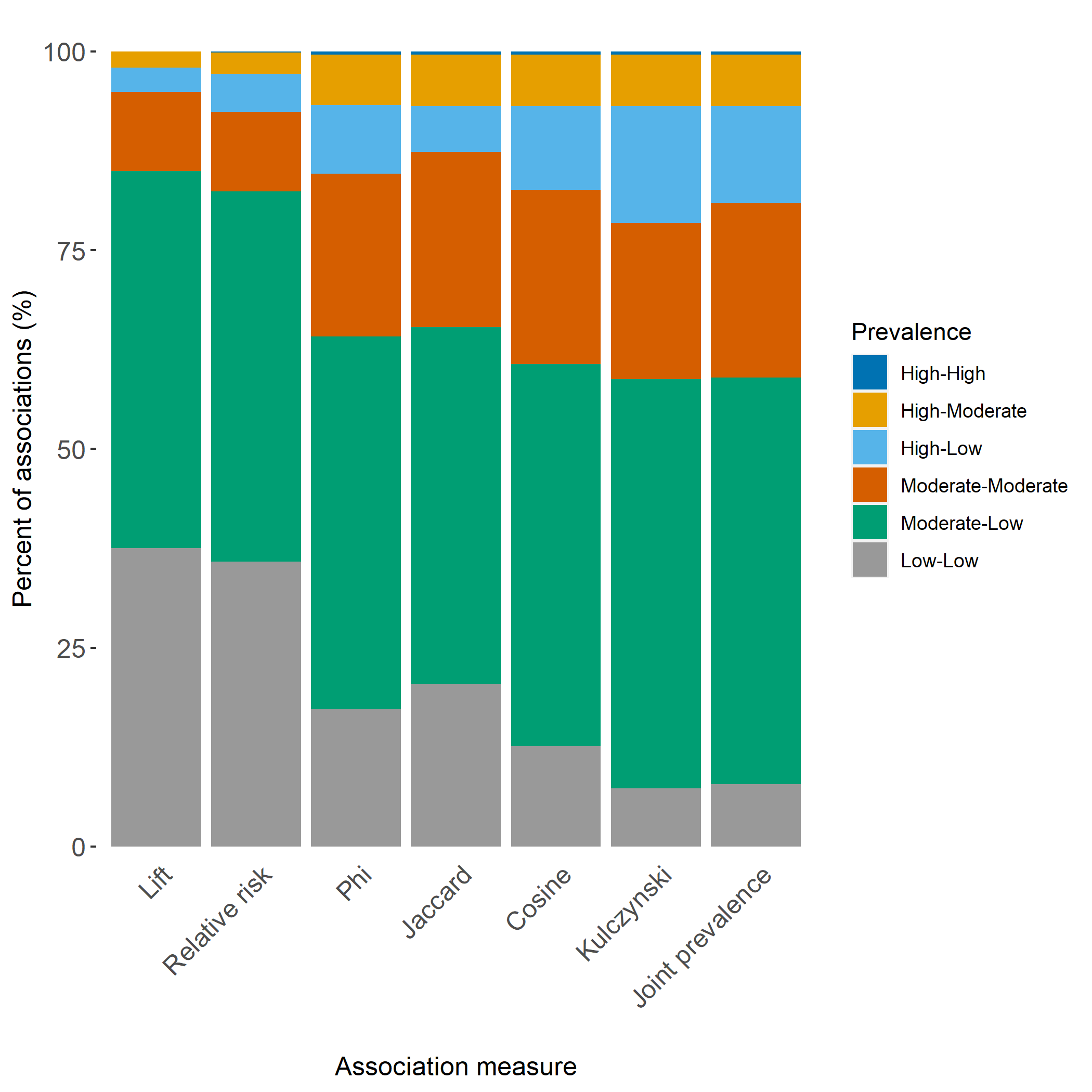


Prevalence was categorized as low (<1%), moderate (1 to <7%), and high (≥7%)

Supplementary Figure 8. Prevalence difference distributions for networks limited to the 200 strongest associations (left) and the strongest 50 percent (*n*=3922) of all statistically significant associations (right)

| 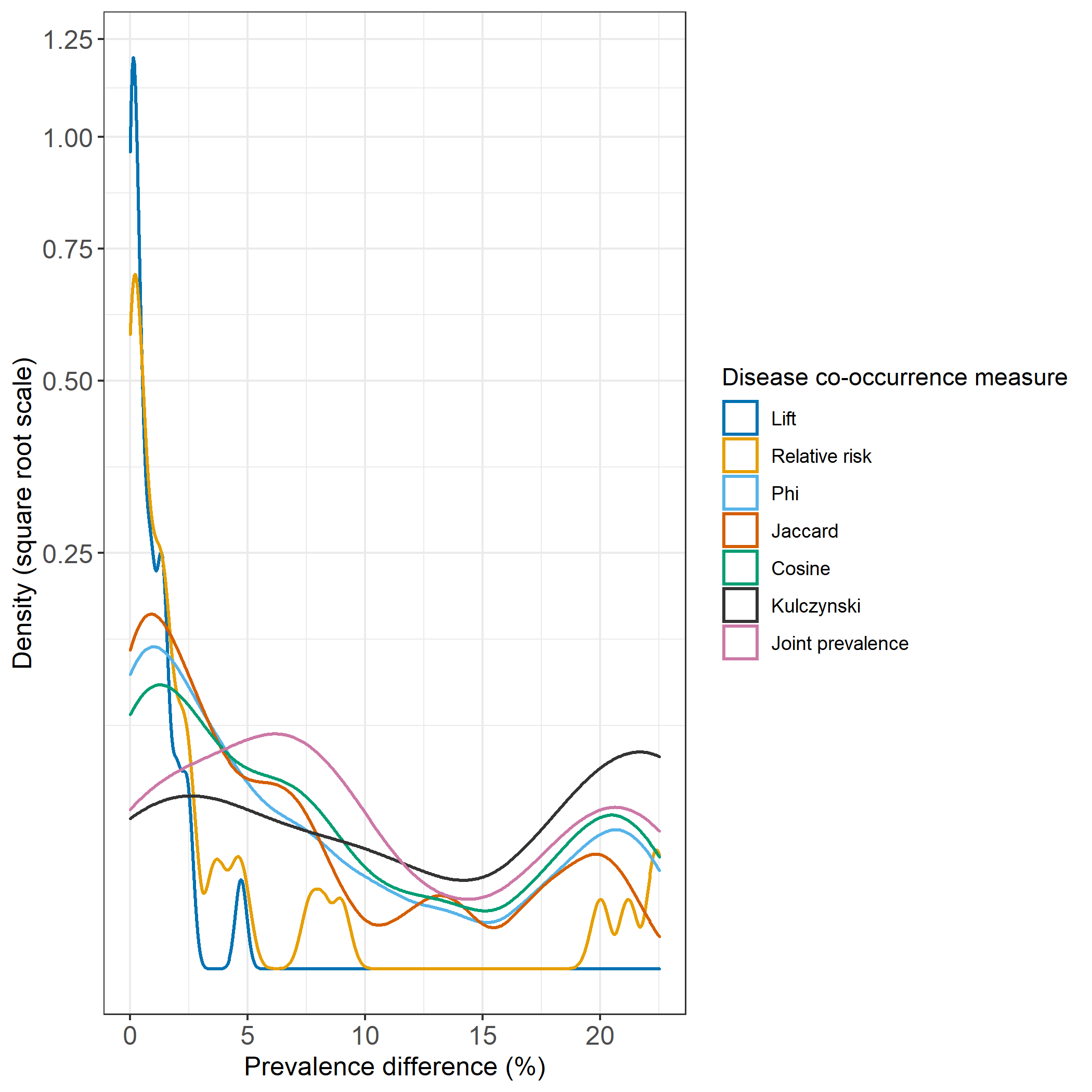 | *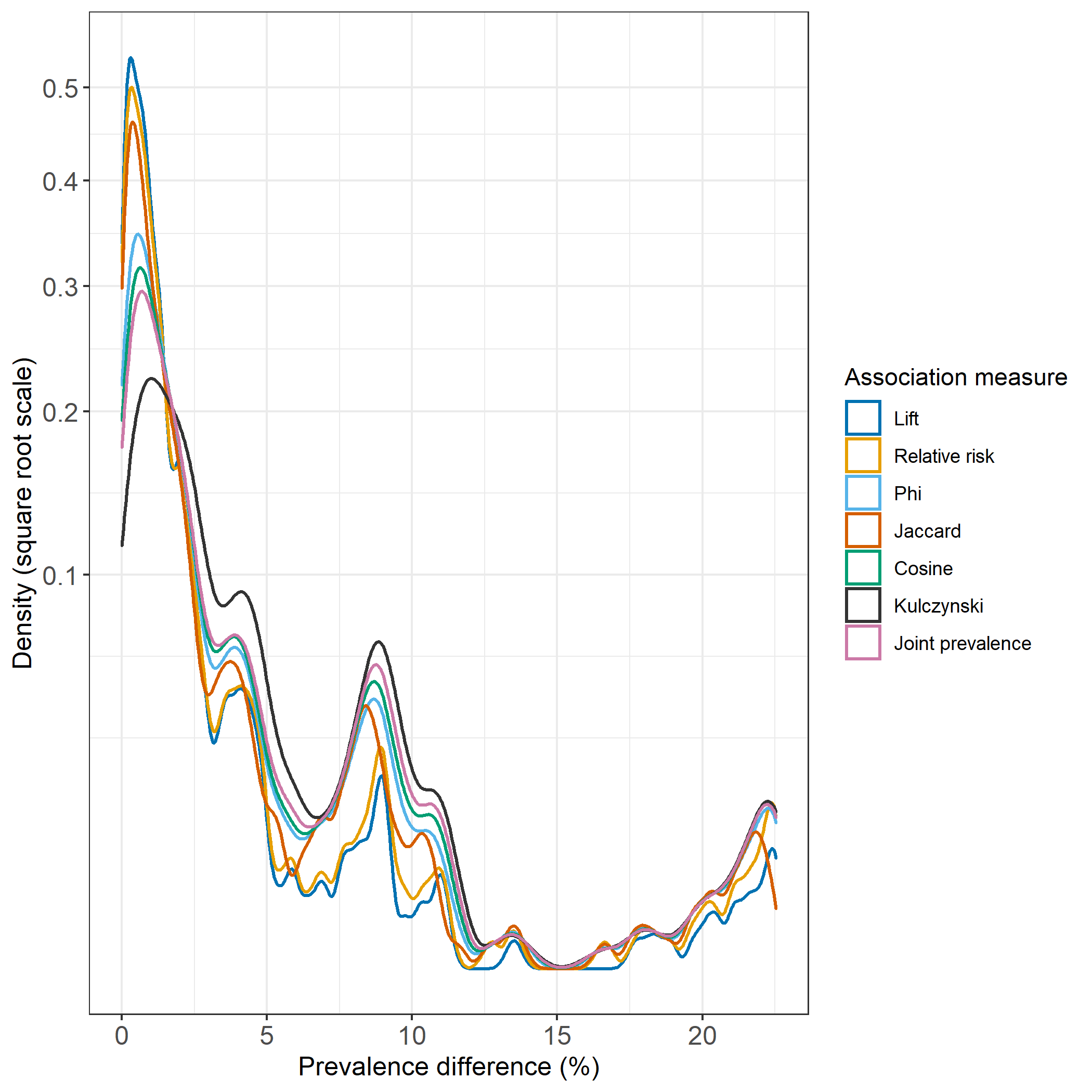* |
| --- | --- |

#

# Supplementary Figure 9. Percent of the 200 strongest associations characterized by prevalence, among select association measures


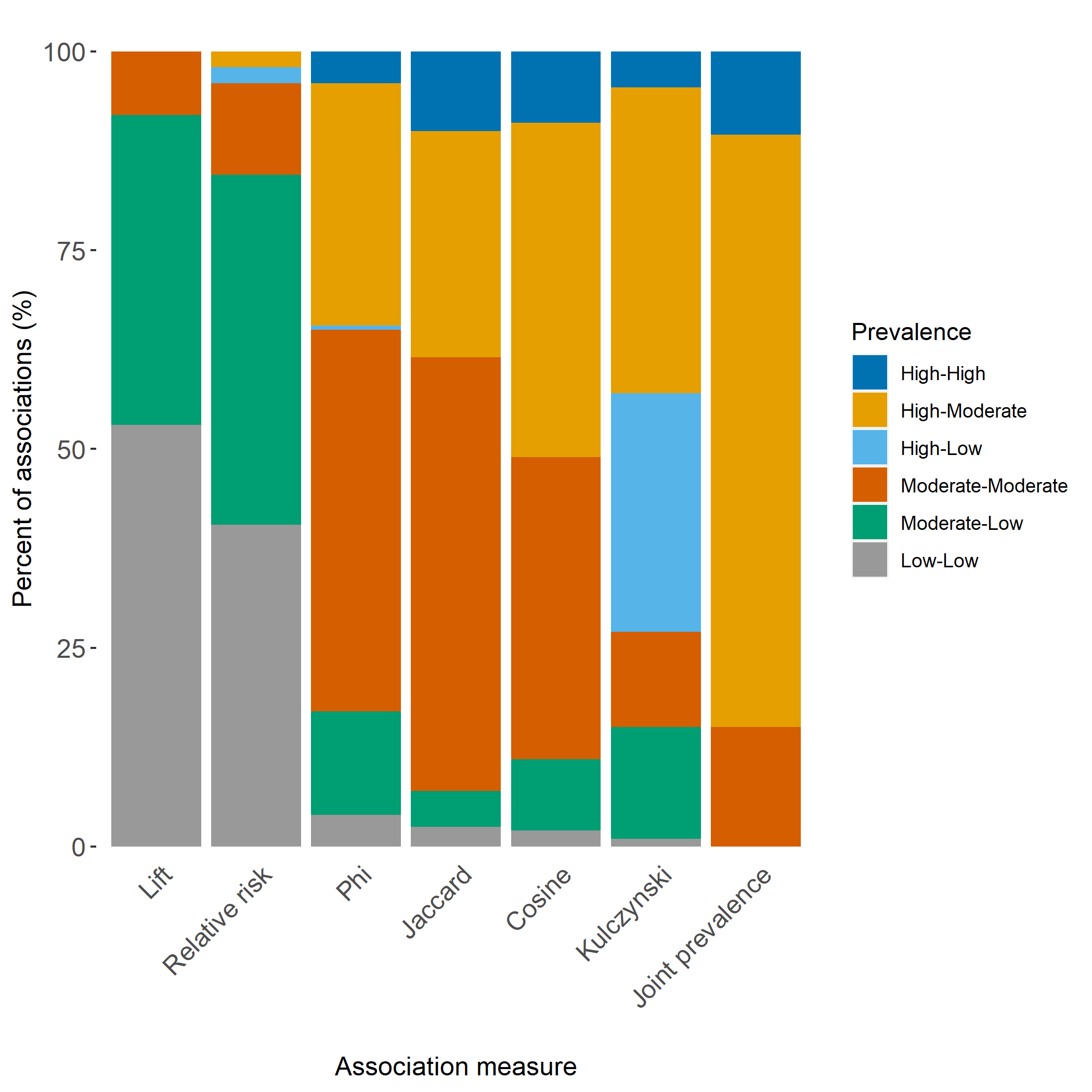


Prevalence was categorized as low (<0.5%), moderate (0.5 to <5%), and high (≥5%)

# Supplementary Figure 10. Percent of the strongest 50 percent (*n*=3922) of all statistically significant associations, characterized by prevalence


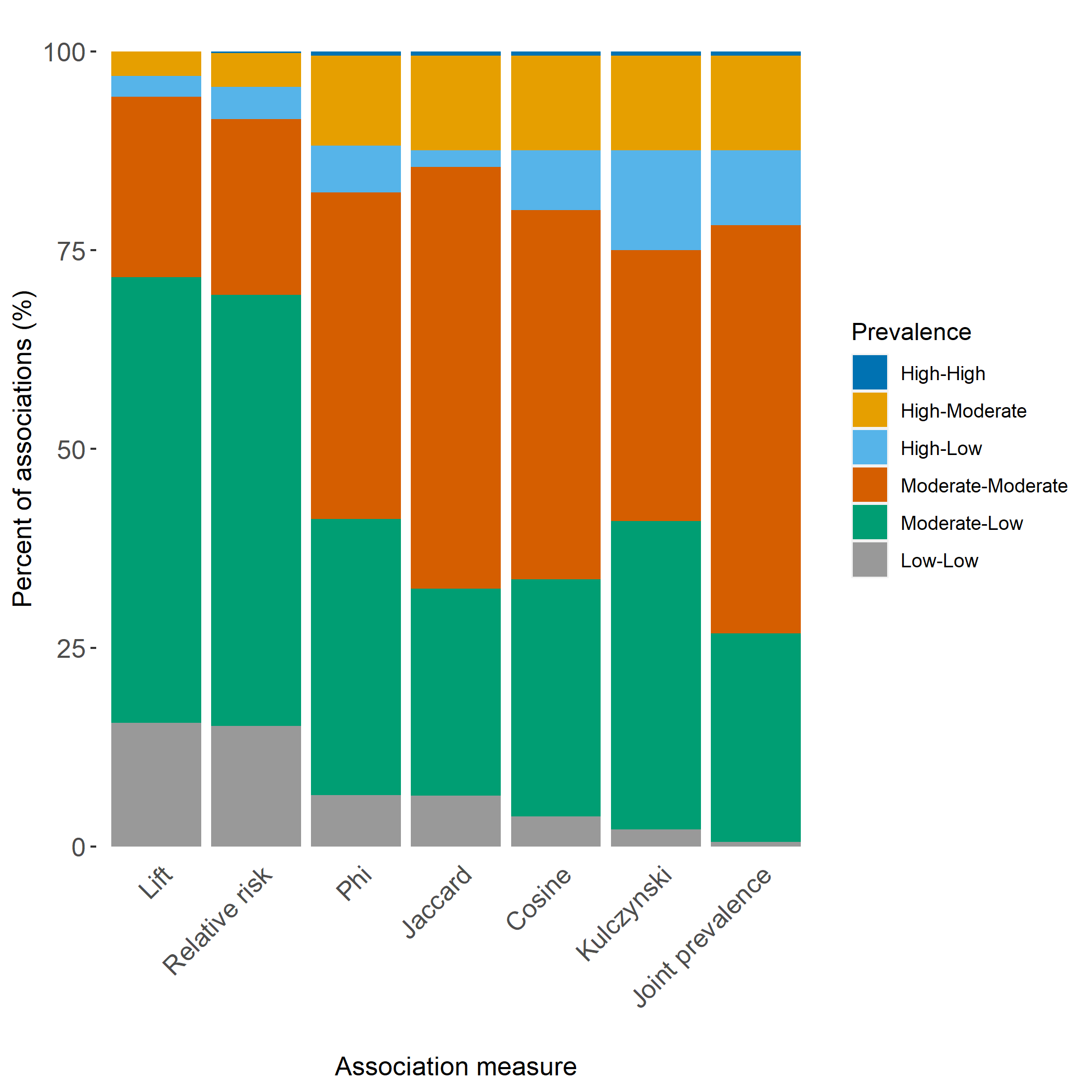


Prevalence was categorized as low (<0.5%), moderate (0.5 to <5%), and high (≥5%)

Supplementary Figure 11. Percent of the strongest 50 percent (*n*=3922) of statistically significant associations in common between networks


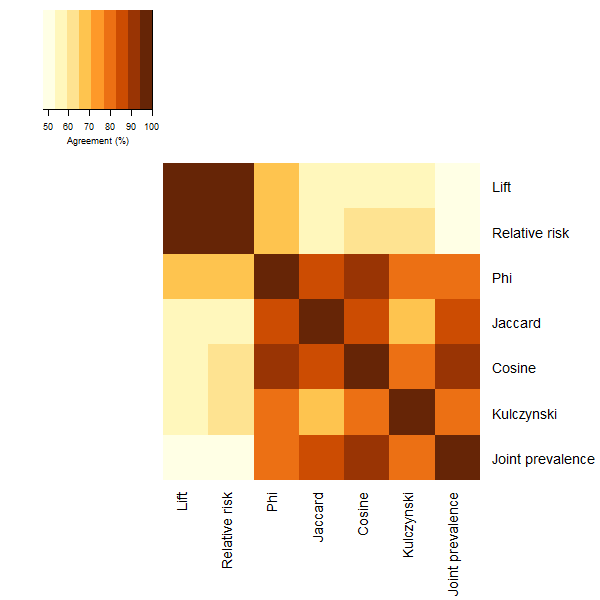


Supplementary Figure 12. Community structure similarity between networks limited to the strongest 50 percent (*n*=3922) of all statistically significant relationships


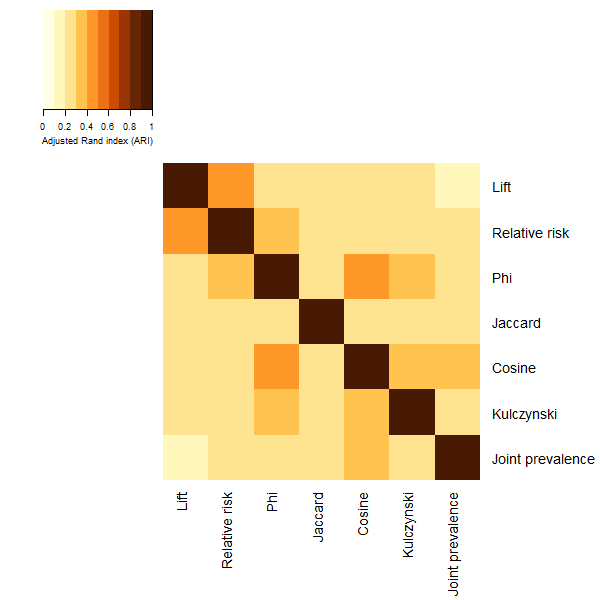


Community structure similarity was measured using the adjusted Rand index (ARI)

Supplementary Figure 13. Agreement on the 20 most central nodes between networks limited to the strongest 50 percent (*n*=3922) of all statistically significant associations


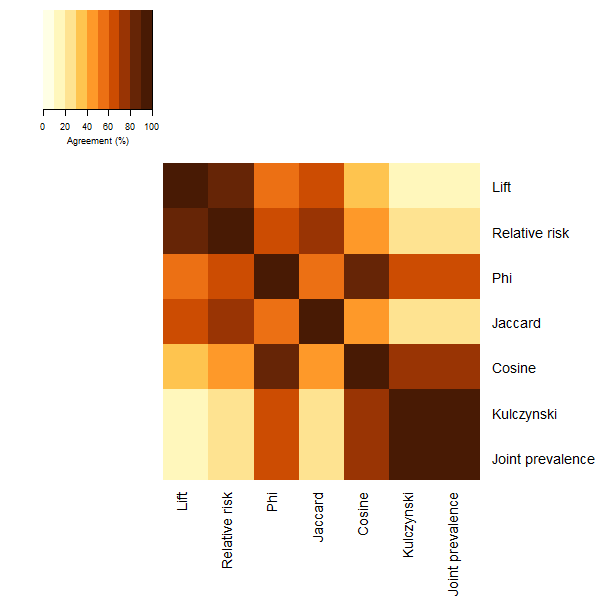


Node centrality was measured using degree centrality

Supplementary Table 1. Top 20 central nodes among networks limited to the 200 strongest associations

|  | Relative risk | Phi | Joint prevalence |
| --- | --- | --- | --- |
| 1 | Aortic aneurysm | Hypertension | Hypertension |
| 2 | Congestive heart failure | Peripheral vascular disease | Type 2 diabetes |
| 3 | Peripheral vascular disease | Congestive heart failure | Degenerative joint disease |
| 4 | ESRD | Ischemic heart disease (excluding AMI) | Disorders of lipid metabolism |
| 5 | Vesicoureteral reflux | Cardiac arrhythmia | Depression |
| 6 | Cerebral palsy | Type 2 diabetes | Ischemic heart disease (excluding AMI) |
| 7 | Cardiomyopathy | Degenerative joint disease | Asthma |
| 8 | Renal disorders, other | Renal disorders, other | Cardiac arrhythmia |
| 9 | Personality disorders | Cataract, aphakia | Hypothyroidism |
| 10 | AMI | Chronic ulcer of the skin | Cataract, aphakia |
| 11 | Quadriplegia and paraplegia | Emphysema, chronic bronchitis, COPD | Congestive heart failure |
| 12 | Cardiac arrest, shock | Dementia | Obesity |
| 13 | Acute respiratory failure | Cerebrovascular disease | Emphysema, chronic bronchitis, COPD |
| 14 | Cardiovascular signs and symptoms | Generalized atherosclerosis | Anxiety, neuroses |
| 15 | Malignant neoplasms, liver and biliary tract | Chronic renal failure | Dementia |
| 16 | Dementia | Depression | Glaucoma |
| 17 | Chronic renal failure | Neurologic disorders, other | Sleep apnea |
| 18 | Malignant neoplasms, stomach | Cardiovascular disorders, other | Other endocrine disorders |
| 19 | Urinary symptoms | Diabetic retinopathy | Deficiency anemias |
| 20 | Hypertension | Disorders of lipid metabolism | Cerebrovascular disease |

Node centrality was measured using degree centrality
AMI = acute myocardial infarction, COPD = chronic obstructive pulmonary disease, ESRD = end-stage renal disease
